# Exploring the link between serum uric acid and colorectal cancer: Insights from genetic evidence and observational data

**DOI:** 10.1097/MD.0000000000040591

**Published:** 2024-11-22

**Authors:** Ying Chen, Shu Zhang, Juanjuan Wu, Di Xu, Cong Wei, Fajiu Li, Guozhu Xie

**Affiliations:** a Department of Radiation Oncology, Nanfang Hospital, Southern Medical University, Guangzhou, China; b Department of Pulmonary and Critical Care Medicine, Affiliated Hospital of Jianghan University, Wuhan, China.

**Keywords:** all-cause mortality, causal association, colorectal cancer, serum urate

## Abstract

Colorectal cancer (CRC) is a major cause of cancer-related mortality worldwide. Urate, known for its antioxidant properties, may influence CRC risk and prognosis, but research on this is limited. We used Mendelian randomization (MR) analysis to explore the causal relationship between serum urate levels and CRC risk. Additionally, we analyzed National Health and Nutrition Examination Survey data to assess the impact of serum urate on CRC prognosis. MR analysis in the European population indicated that higher serum urate levels are associated with a reduced CRC risk (odds ratios [OR] inverse-variance weighted: 0.90, 95% CI: 0.81–0.99, *P* = .04; OR MR-Egger: 0.86, 95% CI: 0.75–0.98, *P* = .03; OR Weighted-Median: 0.85, 95% CI: 0.74–0.96, *P* = .01; OR Weighted-Mode: 0.83, 95% CI: 0.74–0.94, *P* = .002). Validation datasets supported this (OR inverse-variance weighted: 0.83, 95% CI: 0.72–0.96, *P* = .011). However, National Health and Nutrition Examination Survey data showed that higher serum urate levels are linked to poorer CRC outcomes (HR 1.50, 95% CI: 1.08–2.10, *P* = .02). This study suggests that elevated serum urate levels may reduce CRC risk but are associated with worse prognosis in CRC patients, highlighting its potential as a biomarker for CRC risk and prognosis.

**Key points**

- In a pioneering investigation, we explored the complex role of urate in colorectal cancer through MR and data from the NHANES.
- Our MR analysis revealed a causal association between higher serum urate levels and a reduced risk of CRC.
- Findings from the NHANES database showed a significant correlation between elevated serum urate levels and poor outcomes in CRC patients.

## 1. Introduction

Colorectal cancer (CRC) is the third most common cancer in both men and women and ranks as the third leading cause of cancer-related deaths.^[1]^ In 2018 alone, approximately 140,250 new cases of CRC were diagnosed, with an estimated 50,630 deaths resulting from the disease.^[1]^ Most CRC cases (about 70–75%) occur sporadically and are closely linked to modifiable risk factors.^[1–3]^ Research on these risk factors, including smoking, alcohol consumption, physical inactivity, poor diet, and obesity, has significantly advanced our understanding of CRC etiology.^[4]^ Efforts to improve these modifiable risk factors have shown promise in preventing CRC, and early detection of polyps through effective screening interventions has demonstrated potential in reducing mortality rates.^[5]^ However, the impact of residents’ nutritional levels on CRC risk and mortality remains largely unknown as the economy develops.

Urate, the final breakdown product of purine metabolism from both dietary sources and cellular activity, is a potent antioxidant and a major contributor to the overall antioxidant capacity of plasma.^[6]^ Previous epidemiological studies examining the relationship between plasma urate levels and cancer incidence and mortality have yielded conflicting results.^[7–10]^ Some studies found no association,^[8,11]^ while most reported higher plasma urate levels correlated with increased cancer risk and mortality.^[9,10]^ These inconsistent findings may be due to small sample sizes, inadequate adjustment for confounders, use of cross-sectional or case-control designs, or a focus on specific cancer types. Plasma urate levels are influenced by numerous confounders that can distort the association with cancer and overall mortality,^[12]^ and cancer itself can elevate urate concentrations through cancer-related cell death. Therefore, these associations may be subject to confounding or reverse causation. Due to the limitations of observational designs, the true causal relationship between urate levels and CRC risk remains unclear, necessitating new methodologies to overcome these limitations.

Mendelian randomization (MR) has emerged as a powerful tool for assessing causal relationships between exposures and outcomes by using genetic variants as instrumental variables (IVs). MR leverages the principles of genetic inheritance and random assortment of alleles during gamete formation to identify potential causal effects.^[13]^ This methodology offers valuable insights into the possible causal association between urate levels and CRC risk, mitigating biases common in observational studies. Our study aimed to investigate whether there is evidence supporting a causal link between genetically predicted serum urate levels and the risk of developing CRC. Additionally, we explored the influence of serum urate levels on overall mortality in CRC populations, using data from the National Health and Nutrition Examination Survey (NHANES). NHANES is a comprehensive survey that assesses the health and nutritional status of noninstitutionalized individuals. To our knowledge, our study is the first to comprehensively examine the relationship between serum urate levels and CRC mortality using a representative sample of the American population. Understanding the associations between serum urate levels, CRC risk, and mortality will provide valuable insights for preventing the occurrence and progression of CRC.

## 2. Method

### 2.1. Study design

**Figure 1.**
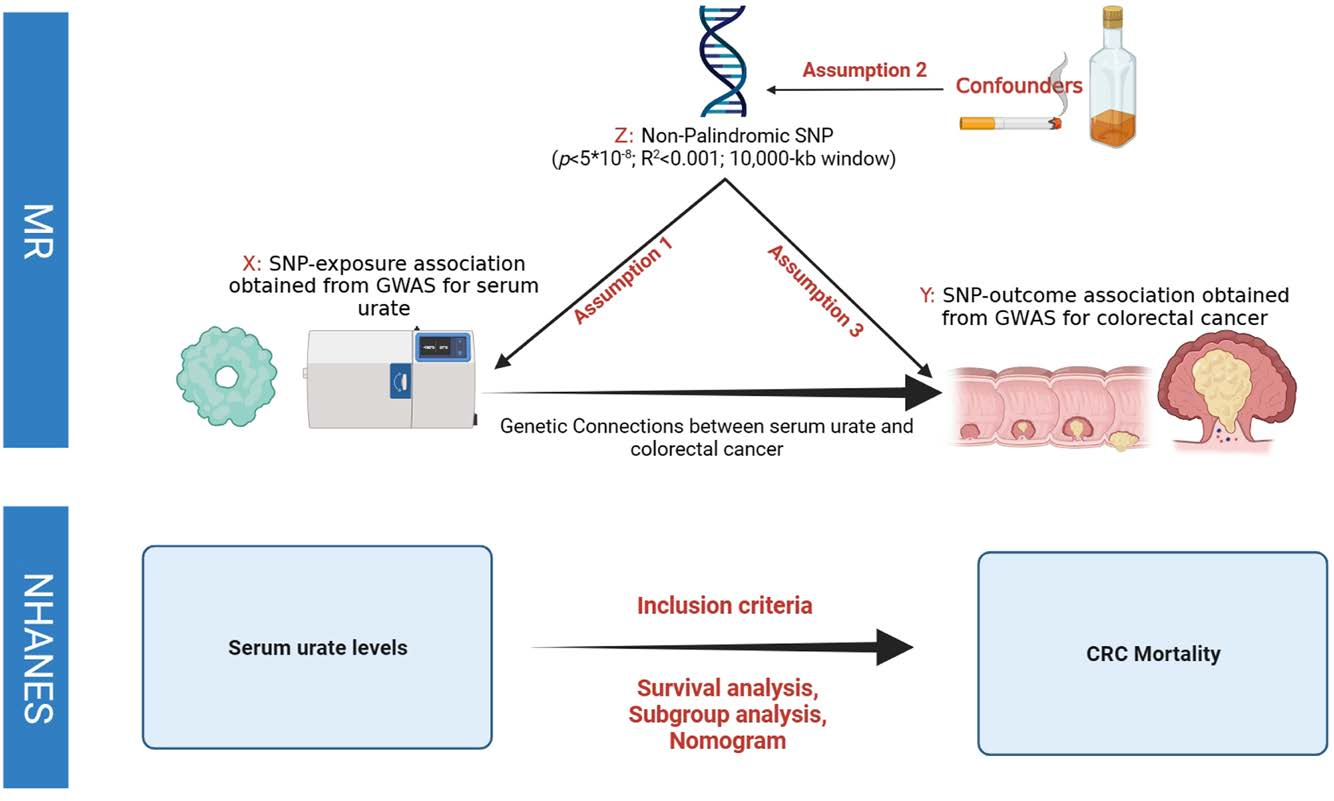
Overview of the present study.

Figure 1 presents the schematic of our study design. We conducted a MR analysis to investigate the causal relationship between serum urate levels and CRC risk. Additionally, data from the NHANES were utilized to assess the impact of serum urate on CRC mortality. The fundamental MR assumptions are: (a) the genetic variant is strongly associated with serum urate, (b) it is not linked to confounders that could bias the serum urate-CRC relationship, and (c) it influences CRC risk solely through its effect on serum urate (Fig. 1). No ethics approval or written consent was required for this secondary analysis of public data.

### 2.2. Selection of IVs

To rigorously adhere to these principles, we implemented 4 criteria for selecting IVs. First, we identified single nucleotide polymorphisms (SNPs) from genome-wide association studies (GWASs) that showed a significant correlation with both serum urate and CRC (*P* < 5 × 10^−8^). Next, we performed linkage disequilibrium clumping with an *R*^2^ threshold of < 0.001 and a 10,000 kb window to retain SNPs with the strongest associations with serum urate. Third, we used the PhenoScanner database to identify any additional traits or conditions influenced by the selected SNPs to address potential pleiotropy. Finally, we excluded palindromic SNPs to mitigate potential pleiotropic effects and enhance the validity of our analysis. To assess the strength of the selected SNPs, we calculated the F-statistics for each instrument. F-statistics above 10 are generally considered indicative of strong instruments that are unlikely to suffer from weak instrument bias. In our analysis, all selected SNPs exhibited F-statistics well above this threshold (F-statistic: 1134), confirming the adequacy of our instruments and reducing the likelihood of bias due to weak instruments.

### 2.3. Study population in MR and NHANES

The summary statistics for serum urate were obtained from two large-scale GWASs. The first GWAS, conducted by the Neale lab, included 13,585,994 genetic variants. The second GWAS used data from the UK Biobank, involving 389,404 participants.^[14]^ For CRC analysis, we extracted genetic variants from a study with 19,948 cases and 12,124 controls from the UK Biobank^[15]^ (Table 1). All participants in the MR analysis were of European descent.

**Table 1:** Details of GWASs in the present study.

Trait	Consortium or first author	Number of variants	GWAS trait ID	Ethnicity	Year
*Exposure*					
Urate (Internal dataset)	Neale lab	13,585,994	ukb-d-30880_irnt	European	2018
Urate (External dataset)	UK Biobank	10,783,684	ebi-a-GCST90014015	European	2021
*Outcome*					
Colorectal cancer	UK Biobank	38,356,021	ebi-a-GCST012879	European	2018
*Confounders*					
Smoking initiation	Liu M	11,802,365	ieu-b-4877	European	2019
Alcohol drinker	Neale lab	13,586,591	ukb-d-20117_2	European	2018

GWAS = genome wide association study.

We used NHANES data from 10 cycles spanning 1999 to 2016 (1999–2000, 2001–2002, 2003–2004, 2005–2006, 2007–2008, 2009–2010, 2011–2012, 2013–2014, and 2015–2016). Serum urate levels were measured using the DxC800 synchron with a timed endpoint method. Covariates included in our analysis were sex, ethnicity, education level, alcohol consumption, smoking behavior, and poverty-to-income ratio.

### 2.4. Statistical analysis

We primarily used the inverse-variance weighted (IVW) method for the main analysis. Additionally, we employed alternative MR techniques such as the weighted median estimator, weighted mode, simple mode, and MR-Egger regression to assess potential causal relationships using the^[16,17]^ using the TwosampleMR package in R software (Version 4.2.0). The statistical models considered potential confounding variables, including smoking and alcohol consumption, while addressing pleiotropy. Results from the MR analysis were presented as odds ratios (ORs) and hazard ratios (HRs), with 95% confidence intervals (CIs). We evaluated the validity of MR assumptions, including the absence of pleiotropy and heterogeneity, using several methods. The funnel plot assessed potential heterogeneity, and MR-Egger intercept regression identified and corrected for potential pleiotropic effects. This regression technique differs from conventional MR methods by allowing the intercept to deviate from 0, which serves as a direct test for the presence of directional pleiotropy among the IVs. If the intercept from the MR-Egger regression is significantly different from 0, it indicates the presence of pleiotropic effects that may bias the MR estimates.^[17,18]^ To ensure robustness and mitigate random errors from IVs, we used the leave-one-out approach and Mendelian Randomization Pleiotropy RESidual Sum and Outlier (MR-PRESSO) method to evaluate the influence of specific SNPs on significant outcomes in the IVW analysis. The MR-PRESSO test operates by identifying outlier SNPs whose effects on the outcome are disproportionately large compared to their effects on the exposure, indicative of potential pleiotropic pathways. Once identified, these outliers are excluded, and the causal estimate is recalculated using the remaining SNPs. The MR-PRESSO test not only identifies but also corrects for pleiotropy, thereby refining the estimate of the causal relationship.^[19]^ Differences in overall survival between the urate-high and urate-low groups were assessed using Kaplan–Meier analysis with the log-rank method. We constructed a prognostic nomogram incorporating serum urate and clinical characteristics using the “Regression Modeling Strategies” R package. We performed a propensity score matched analysis to infer the effects of serum urate on overall survival among patients with CRC. Briefly, we used logistic regression to calculate the propensity score of with confounding variables including age, gender, race, smoking history, and family history of cancer.

## 3. Results

**Figure 2.**
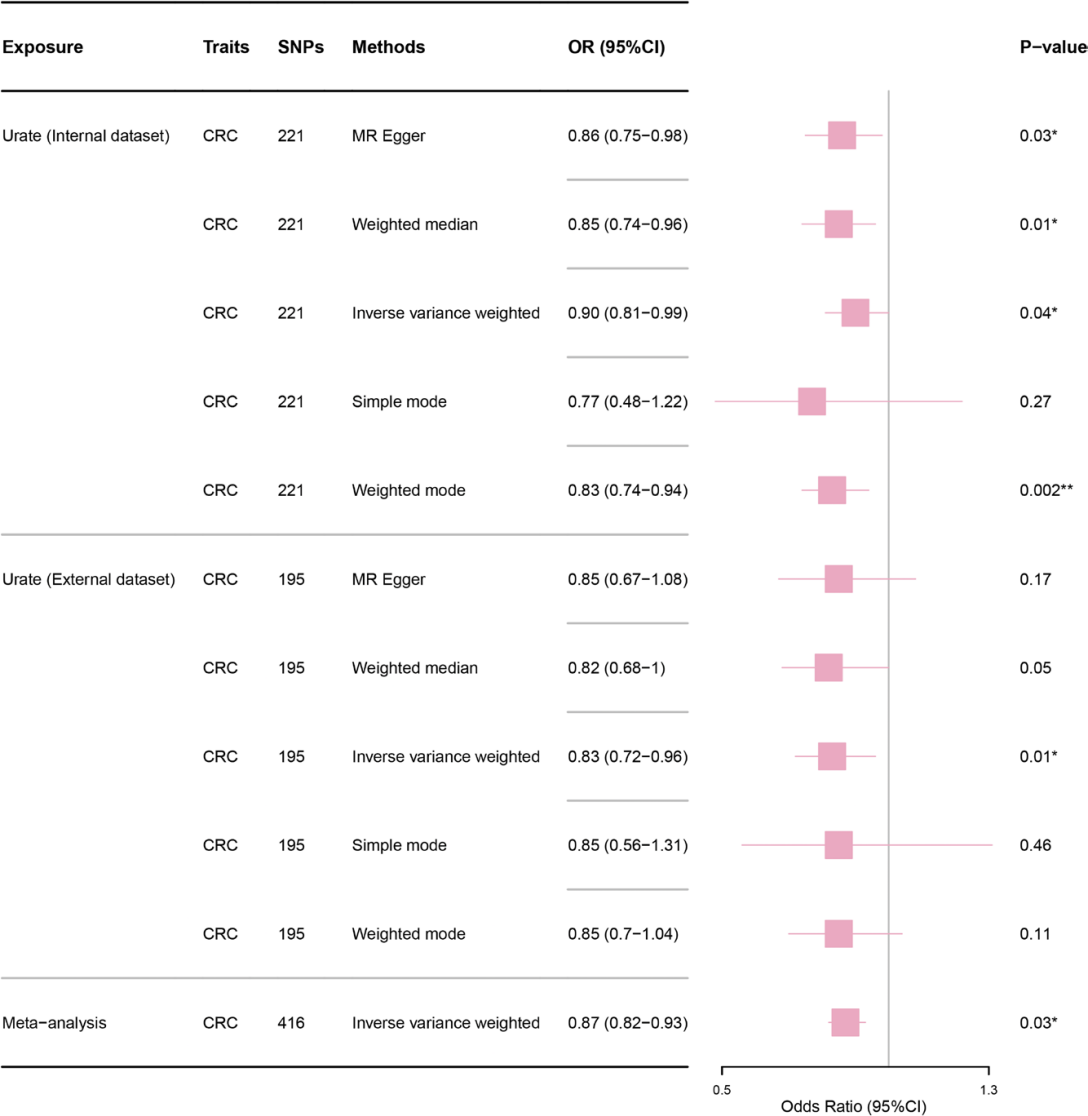
Associations between serum urate and colorectal cancer according to five MR methods. IVW = inverse-variance weighted, SNPs = single nucleotide polymorphisms.

Figure 2 illustrates the IVs estimation results from our study. The MR analysis revealed a significant negative association between serum urate and CRC risk (OR _IVW_: 0.90, 95% CI: 0.81–0.99, *P* = .04; OR _MR-Egger_: 0.86, 95% CI: 0.75–0.98, *P* = .03; OR _Weighted-Median_: 0.85, 95% CI: 0.74–0.96, *P* = .01; OR _Weighted-mode_: 0.83, 95% CI: 0.74–0.94, *P* = .002). Consistent results from the validation dataset further support that serum urate levels may causally reduce CRC risk (OR _IVW_: 0.83, 95% CI: 0.72–0.96, *P* = .011). A meta-analysis of both internal and external datasets confirmed the causal relationship between serum urate levels and CRC risk (OR: 0.87, 95% CI: 0.82–0.93, *P* = .03).

**Figure 3.**
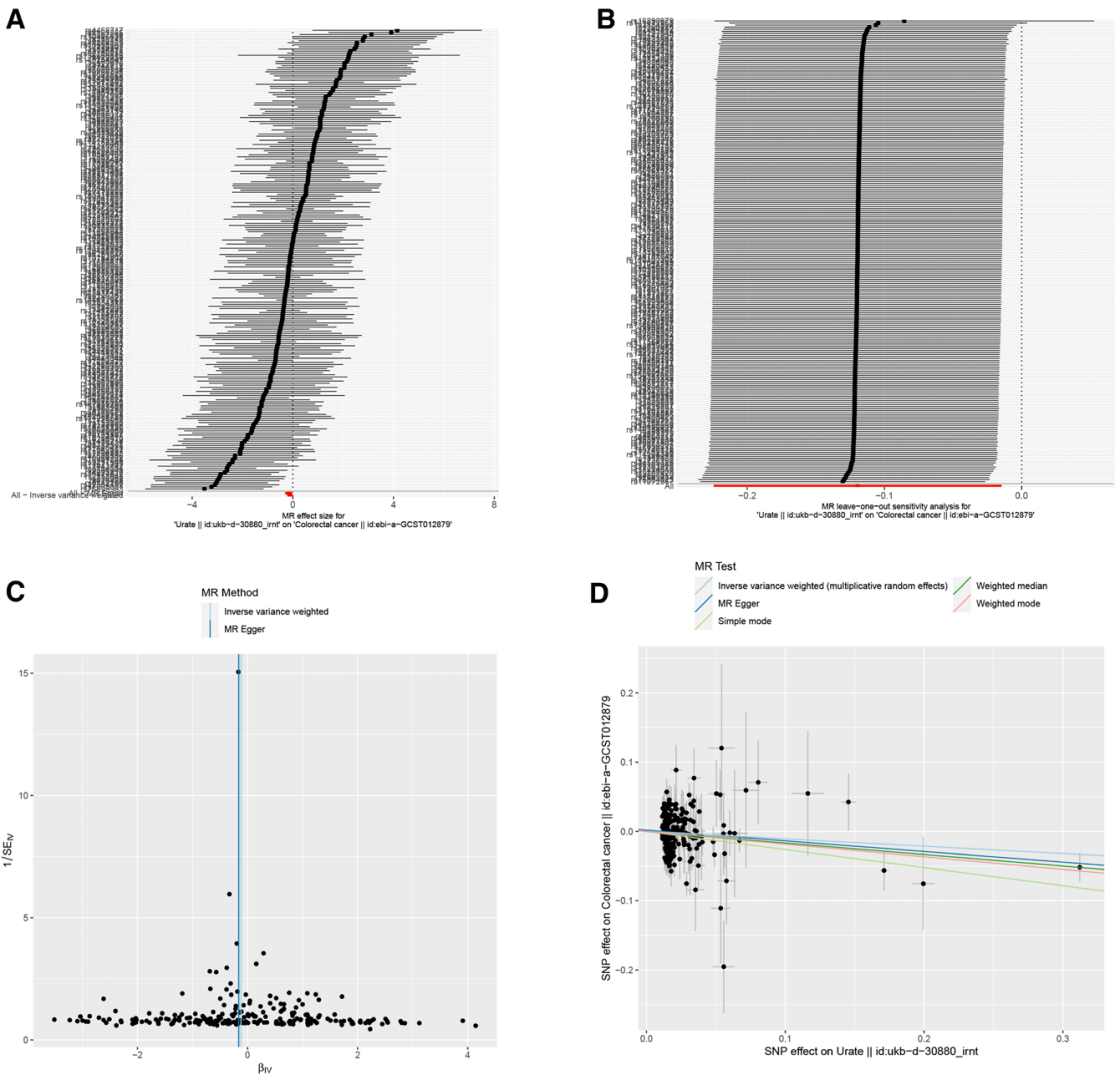
Results of overall effects, leave-one-out analysis, funnel plot, and scatter plot. (A) MR estimate of inverse-variance weighted method. (B) Leave-one-out analysis. (C) Funnel plot. (D) Scatter plot. In the graph, the x-axis portrays the β-estimate derived from a preceding publication, elucidating the correlation amidst each single nucleotide polymorphism (SNP) and the aforementioned exposures that are currently being examined. Conversely, the y-axis exemplifies the β-estimate acquired from a multivariate logistic regression model, effectively representing the intricate nexus connecting each SNP and the peril of developing cancer. Comprehensively manifested on the graph are the causal estimates derived from an assorted collection of Mendelian randomization methods. The inclination of each line accurately delineates the estimated effect in accordance with the specific technique employed. The dots adorning the graph aptly epitomize the observed marginal genetic associations between both the exposures and the risk of the outcome for each SNP variant. Furthermore, the error bars conspicuously illustrate the 95% confidence intervals, while the term “SD” corresponds to the standard error, furnishing additional insights into the precision of the data. MR = Mendelian randomization.

The current investigation employed sensitivity analyses to validate the initial MR findings, ensuring adherence to the 3 fundamental assumptions of MR (as described in Section 2). The outcomes of the sensitivity analyses are presented in Figure 3. Firstly, we identified 221 SNPs associated with both serum urate levels and CRC at a significance level of *P* < 5.0 × 10^−8^, after removing unqualified SNPs (Table S1, Supplemental Digital Content, http://links.lww.com/MD/O13, Fig. 3A). Secondly, employing the leave-one-out technique, we observed that no single SNP significantly influenced the overall effect of serum urate on CRC (Fig. 3B). Furthermore, the funnel plot demonstrated no substantial heterogeneity among the studies (Fig. 3C), and all MR approaches consistently indicated the same directions of effect (Fig. 3D). Notably, the MR-Egger intercept regression yielded mostly nonsignificant *P*-values, suggesting the absence of substantial heterogeneity and horizontal pleiotropy, respectively (Egger regression intercept: 0.0012, standard error: 0.0023, directionality *P*-value: .595). Even after excluding the suspected pleiotropic SNPs (rs3184504), our MR-PRESSO findings remained consistent with the original results obtained using the set of IVs (beta-value: −0.106, standard error: 0.05, *P* = .04). These findings provide support for the adherence to the second MR assumption. Additionally, with regard to the third assumption, we investigated potential connections between confounding factors (smoking and alcohol) and both the exposure (serum urate) and the outcome (CRC). As a result, we found no evidence of causal associations between serum urate and smoking (OR 1.01, 95% CI 0.98–1.04, *P* = .40), nor between serum urate and alcohol (OR 1.0002, 95% CI 0.9966–1.0038, *P* = .90). These results suggest that the third assumption of MR was not violated.

**Figure 4.**
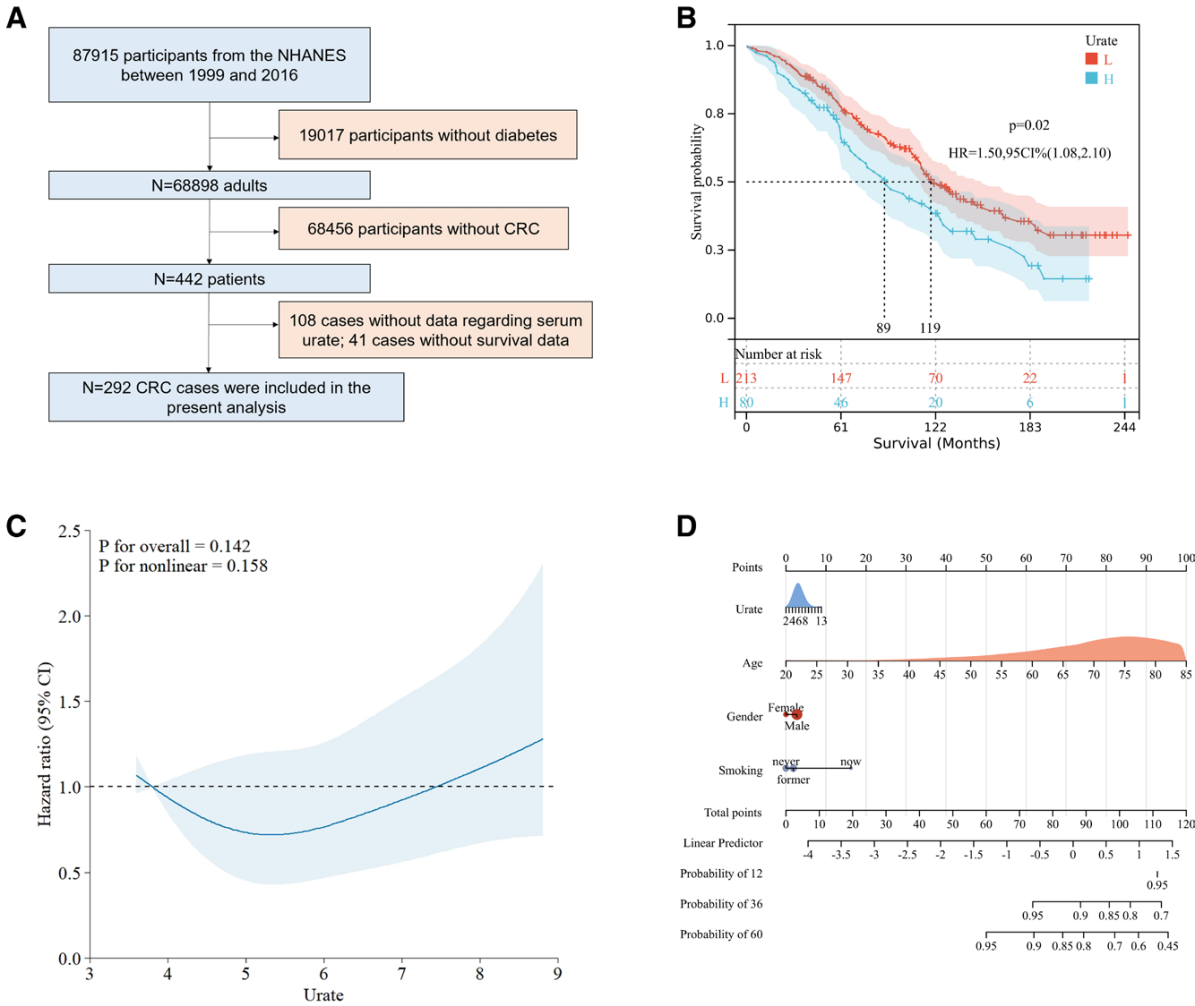
Results of NHANES analysis. (A) Inclusion criteria. (B) Survival analysis according to serum urate levels. (C) Restricted cubic splines. (D) Nomogram based on serum urate and clinical variables. NHANES = National Health and Nutrition Examination Survey.

Eventually, the results from the NHANES database extended the MR findings. After excluding individuals with missing data on serum urate (exposure) and those without CRC (outcome), a total of 292 CRC cases were included in our analysis (Fig. 4A). Detailed information of included 292 patients were listed in Table S2, Supplemental Digital Content, http://links.lww.com/MD/O14. The survival analysis illustrated high value of serum urate was associated with poor outcomes of patients with CRC (HR 1.50, 95% CI 1.08–2.10, *P* = .02) (Fig. 4B). Similarly, the results of propensity score matched analysis revealed that elevated serum urate levels were associated with diminished overall survival in patients with CRC (HR 2.51, 95% CI 1.39–4.51, *P* = 1.5e−03) (Fig. S1, Supplemental Digital Content, http://links.lww.com/MD/O12). Given potential risk factors, we performed a subgroup analysis to identify interaction effects. The results of subgroup analysis between age, gender, and ethnicity on survival among patients with CRC are listed in Table 2. Apart from interactions between gender as well as education (interaction *P*-values < .05), we found significant results in male (HR 1.19, 95% CI 1.03–1.39, *P* = .02) and non-Hispanic white patients (HR 1.14, 95% CI 1.01–1.30, *P* = .04). Restricted cubic splines showed nonlinear relationships between serum urate and mortality (Fig. 4C). Eventually, we combined serum urate and clinical chrematistics (age, gender, and smoking history) to construct a nomogram in the cohort, which displayed a quantitative approach to generate personalized predictions for CRC patients (Fig. 4D).

**Table 2:** Characteristics of NHANCE cohorts.

Character	HR (95% CI)	*P*-value	*P* for interaction
*Gender*			.03
Male	1.19 (1.03, 1.39)	.02*	
Female	0.89 (0.73, 1.10)	.28	
*Age*			.63
≥75	1.12 (0.92, 1.37)	.26	
<75	1.07 (0.92, 1.24)	.37	
*Race*			.01
Non-Hispanic Black	0.71 (0.48, 1.04)	.08	
Non-Hispanic White	1.14 (1.01, 1.30)	.04*	
Mexican American	0.86 (0.41, 1.80)	.69	
Other Hispanic	1.04 (0.42, 2.56)	.93	
Other race – including multiracial	1.16 (0.45, 2.97)	.76	
*Education*			.02
9–11th Grade (includes 12th grade with no diploma)	1.25 (0.91, 1.72)	.17	
College Graduate or above	1.28 (0.76, 2.14)	.35	
<9th Grade	1.07 (0.83, 1.39)	.58	
Some College or AA degree	1.15 (0.87, 1.52)	.31	
High School Grad/GED or Equivalent	0.87 (0.64, 1.18)	.37	
*Smoking*			.11
Never	1.19 (0.98, 1.45)	.09	
Former	0.97 (0.82, 1.16)	.77	
Now	1.17 (0.85, 1.61)	.33	

*means statistical significance.

## 4. Discussion

Our study employed MR to establish a causal relationship between low serum urate levels and increased susceptibility to CRC. Previous research has extensively examined the association between urate and cancer incidence and prognosis,^[7–9]^ yet limited attention has been given to exploring the connection between serum urate levels and CRC incidence and mortality. By elucidating this association, our study is the first to propose a relationship between serum urate and CRC incidence, offering novel insights and potential screening approaches for high-risk individuals. Additionally, we used data from the NHANES database to validate the important role of serum urate in CRC prognosis, providing new understanding and potential implications for future clinical practices.

The relationship between serum urate levels and cancer risk is an intricate and rapidly evolving field of investigation. In parallel with our observations, a recent meta-analysis comprising 1,081,358 participants demonstrated that elevated serum urate levels are associated with an increased risk of CRC, suggesting that maintaining lower urate levels may be advantageous for reducing CRC incidence.^[20]^ Moreover, a retrospective cross-sectional study indicated a substantial correlation between elevated serum uric acid levels and multiple metastatic sites, suggesting that high serum uric acid levels measured at the time of diagnosis in metastatic CRC serve as an accessible and cost-effective biomarker.^[21]^ Several mechanisms may explain the link between high serum urate levels and a lower risk of CRC. One possible mechanism is the antioxidant and anti-inflammatory effects of urate, which contribute to the body’s overall antioxidant capacity.^[22]^ Chronic inflammation and oxidative stress are known to contribute to cancer development and progression.^[23,24]^ Higher urate levels may enhance the body’s antioxidant defenses, reducing DNA damage caused by reactive oxygen species (ROS)^[25,26]^ and dampening chronic inflammation.^[27]^ ROS have been implicated in the induction of DNA damage, the stimulation of cellular proliferation, and the facilitation of epithelial-to-mesenchymal transition, thereby exacerbating tumorigenesis and tumor progression.^[26]^ Additionally, ROS has been correlated with the pathogenesis and advancement of CRC, suggesting a significant role for ROS in oncogenic processes.^[28]^ Another possible mechanism is related to the immune response. Urate can modulate immune responses and activate immune cells, potentially enhancing the immune system’s ability to recognize and eliminate cancer cells.^[29,30]^ A robust immune response is crucial for effective tumor surveillance and suppression. Elevated urate levels might enhance immune activity against CRC cells, leading to a lower risk of CRC. While our findings indicate that elevated urate levels are associated with a reduced risk of developing CRC, they also suggest a complex relationship where serum urate levels correlate with poorer outcomes in CRC patients. This duality presents a unique challenge in understanding urate’s role across different stages of CRC. Firstly, although high urate levels may initiate an overly active immune response, an excessively active immune response can have unintended consequences and potentially contribute to increased all-cause mortality. Specifically, elevated urate levels or urate crystal can induce the production of pro-inflammatory cytokines such as IL-6 and tumor necrosis factor-alpha, which have been associated with cancer progression and poor prognosis in CRC.^[31,32]^ Moreover, a long-term chronic inflammation may lead to the exhaustion of CD8+ T cells, where the immune system becomes less effective at recognizing and attacking cancer cells, thereby contributing to tumor progression and increased all-cause mortality.^[33]^ Secondly, elevated serum urate levels can serve as a biomarker for underlying health conditions.^[34–36]^ Higher urate levels have been associated with conditions such as chronic kidney disease^[35]^ and cardiovascular disease,^[36]^ which themselves increase the risk of all-cause mortality. The association between high urate levels and increased all-cause mortality may be mediated through these comorbid health conditions. Thirdly, differences in uric acid metabolism, influenced by genetic and environmental factors, can result in individual variations in serum urate levels. Individuals with higher urate levels may have this regression technique inherent genetic or metabolic factors that contribute to a lower risk of CRC but higher susceptibility to other causes of mortality.

The primary strength of our study lies in the utilization of MR analyses combined with an observational study design within the NHANES dataset. MR analyses mitigate potential biases, enabling us to ascertain causal associations between serum urate levels and CRC risk. Additionally, the substantial sample size available in NHANES provided valuable support for considering serum urate factors as covariates, allowing us to estimate their impact on CRC mortality. This synergistic integration of MR analyses and observational study effectively elucidated the dual role of urate. Given the potential of urate as a biomarker, we recommend its incorporation into existing clinical guidelines for CRC screening. Urate testing could be introduced as part of routine metabolic panels during regular health assessments, particularly in populations at higher risk for CRC. For individuals with elevated urate levels, a more frequent or earlier screening for CRC using colonoscopy might be justified, given their potentially higher risk of adverse outcomes. Moreover, integrating urate levels into the risk assessment models could help clinicians identify patients who may benefit from aggressive therapeutic strategies or closer monitoring during follow-up. Particular attention should be paid to patient populations with high urate levels and co-existing metabolic syndrome, as these individuals often exhibit multiple risk factors for CRC, including obesity, hypertension, and insulin resistance. Nonetheless, our study, while providing valuable insights, is not without limitations, which must be duly acknowledged. Firstly, despite employing multiple MR methods for identifying and evaluating abnormal genetic variants, we cannot entirely exclude the influence of mediators or pleiotropy. Additionally, the modest ORs observed in our MR analysis suggest a decreased risk of CRC development with higher serum urate levels, with an OR of approximately 0.90. Similarly, the HRs indicate a 50% increase in risk of poorer outcomes in CRC patients with elevated serum urate levels. Although these effect sizes are modest, they can have substantial implications in a public health context. Even a small reduction in CRC risk across a large population could result in significant decreases in CRC incidence, given the high prevalence of the disease. Secondly, our MR analysis was conducted primarily using data from populations of European descent. While this approach provided a robust set of data, it inherently limits the generalizability of our findings to other ethnic groups. Notably, genetic determinants of serum uric acid levels and their associations with CRC risk may vary significantly across different populations due to genetic diversity and environmental interactions. For example, certain alleles associated with urate metabolism have varied frequencies in non-European populations, which may lead to different patterns of disease susceptibility and progression. Additionally, environmental factors such as diet, lifestyle, and exposure to risk factors, which differ markedly across geographical and cultural contexts, can interact with genetic predispositions to influence both urate levels and CRC outcomes. Given these considerations, our findings highlight the need for further studies that include diverse ethnic groups, which not only enhances our understanding of the biological mechanisms underpinning these associations but also improves the development of targeted prevention that are culturally and genetically tailored to diverse populations. Thirdly, as a nutrition-focused database, NHANES has inherent limitations pertaining to the sample size of patients diagnosed with CRC. The absence of detailed CRC staging data limits our ability to precisely stratify patients based on disease severity, potentially conflating outcomes across varied stages of cancer. Similarly, without subtype information, the effect of urate on different pathological forms of CRC, which might respond differently to the metabolic environment influenced by urate, remains unexplored. Furthermore, treatment modalities, which can vary widely in intensity and effectiveness, also play a crucial role in patient prognosis and could interact with urate levels to influence outcomes.

## 5. Conclusion

Our study contributes to the existing evidence on the association between serum urate levels and CRC, highlighting the potential of serum urate as a biomarker for assessing CRC risk. These findings aim to inspire further investigations into the mechanisms by which serum urate impacts CRC, guiding the development of innovative strategies for preventing and managing this condition. Nevertheless, additional research is required to elucidate the complex mechanisms involved in this association.

## Acknowledgments

We appreciate the work of the open GWAS project (https://gwas.mrcieu.ac.uk/).

## Author contributions

**Conceptualization:** Ying Chen, Shu Zhang, Juanjuan Wu, Di Xu, Guozhu Xie.

**Data curation:** Ying Chen, Shu Zhang, Guozhu Xie.

**Formal analysis:** Ying Chen, Shu Zhang.

**Investigation:** Ying Chen.

**Methodology:** Ying Chen, Di Xu.

**Project administration:** Ying Chen.

**Resources:** Ying Chen.

**Software:** Ying Chen, Juanjuan Wu, Guozhu Xie.

**Supervision:** Ying Chen, Di Xu.

**Validation:** Ying Chen.

**Visualization:** Ying Chen, Cong Wei, Fajiu Li.

**Writing – original draft:** Ying Chen, Guozhu Xie.

**Writing – review & editing:** Ying Chen, Guozhu Xie.

## Supplementary Material

SUPPLEMENTARY MATERIAL
