# Supplementary material for: Exploring the link between serum uric acid and colorectal cancer: Insights from genetic evidence and observational data
Source: Medicine (Baltimore). 2024 Nov 22;103(47):e40591. doi: 10.1097/MD.0000000000040591 (PMC11596604; doi:10.1097/MD.0000000000040591)
Supplement: SUPPLEMENTARY MATERIAL [file medi-103-e40591-s001.docx]

| **Supplementary Table S1. Detailed information of SNPs.** | | | | | | | | | | | | | | | | | | | |
| --- | --- | --- | --- | --- | --- | --- | --- | --- | --- | --- | --- | --- | --- | --- | --- | --- | --- | --- | --- |
|  | **SNP** | **effect_allele.exposure** | **other_allele.exposure** | **effect_allele.outcome** | **other_allele.outcome** | **beta.exposure** | **beta.outcome** | **eaf.exposure** | **eaf.outcome** | **chr** | **pos** | **se.outcome** | **samplesize.outcome** | **pval.outcome** | **pos.exposure** | **pval.exposure** | **chr.exposure** | **samplesize.exposure** | **se.exposure** |
| 1 | rs10060524 | G | A | G | A | -0.012212 | 0.0015947 | NA | 0.475166 | 5 | 68696850 | 0.017117 | 32072 | 0.925772 | 68696850 | 7.10E-12 | 5 | 389404 | 0.0017814 |
| 2 | rs10064782 | G | A | G | A | 0.0159891 | -0.035284 | NA | 0.275721 | 5 | 67715161 | 0.019462 | 32072 | 0.0698377 | 67715161 | 6.71E-15 | 5 | 389404 | 0.0020526 |
| 3 | rs10102749 | C | T | C | T | -0.010608 | 0.0108851 | NA | 0.54993 | 8 | 87407333 | 0.0171379 | 32072 | 0.525333 | 87407333 | 3.12E-09 | 8 | 389404 | 0.0017904 |
| 4 | rs10113411 | C | A | C | A | -0.016072 | -0.040554 | NA | 0.240093 | 8 | 38106777 | 0.020505 | 32072 | 0.0479568 | 38106777 | 6.92E-15 | 8 | 389404 | 0.0020642 |
| 5 | rs10114341 | C | T | C | T | -0.012301 | 0.0258118 | NA | 0.4151 | 9 | 96919182 | 0.0170286 | 32072 | 0.129572 | 96919182 | 6.78E-12 | 9 | 389404 | 0.0017926 |
| 6 | rs10158328 | G | A | G | A | 0.0130107 | -0.014976 | NA | 0.442021 | 1 | 155030557 | 0.0169225 | 32072 | 0.376176 | 155027763 | 3.77E-13 | 1 | 389404 | 0.0017912 |
| 7 | rs10164318 | G | T | G | T | -0.019481 | 0.0351686 | NA | 0.642142 | 19 | 33459298 | 0.018104 | 32072 | 0.0520655 | 33459298 | 8.48E-25 | 19 | 389404 | 0.0018946 |
| 8 | rs10224210 | C | T | C | T | 0.0276496 | -0.01137 | NA | 0.249653 | 7 | 151413194 | 0.0186988 | 32072 | 0.54314 | 151413194 | 2.25E-44 | 7 | 389404 | 0.0019787 |
| 9 | rs10405423 | A | C | A | C | 0.0242166 | 0.015217 | NA | 0.692595 | 19 | 7211311 | 0.018016 | 32072 | 0.398313 | 7211311 | 1.71E-37 | 19 | 389404 | 0.0018924 |
| 10 | rs1045411 | T | C | T | C | -0.013762 | 0.0009943 | NA | 0.223558 | 13 | 31033232 | 0.0203617 | 32072 | 0.961052 | 31033232 | 6.69E-12 | 13 | 389404 | 0.002005 |
| 11 | rs1047891 | A | C | A | C | -0.027221 | -0.011325 | NA | 0.295502 | 2 | 211540507 | 0.018372 | 32072 | 0.537617 | 211540507 | 7.98E-46 | 2 | 389404 | 0.0019157 |
| 12 | rs10507059 | T | C | T | C | 0.0162161 | -0.011315 | NA | 0.187756 | 12 | 96105834 | 0.0207457 | 32072 | 0.585466 | 96105834 | 1.35E-14 | 12 | 389404 | 0.0021057 |
| 13 | rs10782230 | A | G | A | G | 0.0141749 | -0.023343 | NA | 0.486602 | 6 | 126228512 | 0.0169889 | 32072 | 0.169431 | 126228512 | 1.72E-15 | 6 | 389404 | 0.0017808 |
| 14 | rs10808961 | A | G | A | G | -0.012896 | -0.021333 | NA | 0.343374 | 8 | 42334926 | 0.0181787 | 32072 | 0.240585 | 42334926 | 8.34E-12 | 8 | 389404 | 0.0018875 |
| 15 | rs10817881 | G | A | G | A | 0.0105313 | 0.0143723 | NA | 0.618868 | 9 | 119140488 | 0.0185397 | 32072 | 0.438212 | 119140488 | 2.49E-08 | 9 | 389404 | 0.0018893 |
| 16 | rs10846156 | G | T | G | T | -0.015783 | -0.012746 | NA | 0.213829 | 12 | 15321921 | 0.0212529 | 32072 | 0.548698 | 15321921 | 1.15E-12 | 12 | 389404 | 0.0022193 |
| 17 | rs10886117 | A | G | A | G | 0.02013 | 0.0232689 | NA | 0.173923 | 10 | 119480578 | 0.0244198 | 32072 | 0.340654 | 119480578 | 3.75E-17 | 10 | 389404 | 0.0023906 |
| 18 | rs10901057 | G | C | G | C | 0.0200047 | -0.041906 | NA | 0.863767 | 9 | 134245698 | 0.0284293 | 32072 | 0.140471 | 134245698 | 2.29E-09 | 9 | 389404 | 0.0033475 |
| 19 | rs10901812 | A | G | A | G | 0.0177026 | 0.0187859 | NA | 0.238699 | 10 | 126419384 | 0.0201095 | 32072 | 0.350213 | 126419384 | 1.82E-18 | 10 | 389404 | 0.0020191 |
| 20 | rs10949591 | A | G | A | G | 0.0164748 | -0.012632 | NA | 0.785547 | 7 | 156261321 | 0.0223018 | 32072 | 0.571126 | 156261321 | 2.69E-12 | 7 | 389404 | 0.0023559 |
| 21 | rs11072567 | G | A | G | A | 0.0294537 | 0.0526676 | NA | 0.521499 | 15 | 76298744 | 0.0172963 | 32072 | 0.0023266 | 76298744 | 1.09E-61 | 15 | 389404 | 0.0017772 |
| 22 | rs11084211 | A | G | A | G | -0.013509 | 0.0192439 | NA | 0.372739 | 19 | 53460861 | 0.0198964 | 32072 | 0.33344 | 53460861 | 9.82E-13 | 19 | 389404 | 0.0018939 |
| 23 | rs111346856 | A | G | A | G | 0.0147009 | 0.0175199 | NA | 0.214677 | 10 | 65193018 | 0.0199869 | 32072 | 0.38072 | 65193018 | 1.46E-13 | 10 | 389404 | 0.0019892 |
| 24 | rs11136326 | G | A | G | A | 0.0118721 | 0.0008772 | NA | 0.566522 | 8 | 144910239 | 0.0173294 | 32072 | 0.959629 | 144890569 | 7.50E-10 | 8 | 389404 | 0.0019288 |
| 25 | rs111625726 | C | T | C | T | -0.048849 | 0.0148171 | NA | 0.255308 | 4 | 89090354 | 0.0194529 | 32072 | 0.446246 | 89090354 | 4.16E-129 | 4 | 389404 | 0.0020207 |
| 26 | rs11202328 | T | C | T | C | -0.027566 | -0.032897 | NA | 0.18401 | 10 | 88845190 | 0.0274783 | 32072 | 0.231233 | 88845190 | 1.57E-25 | 10 | 389404 | 0.0026395 |
| 27 | rs11205458 | G | C | G | C | 0.014574 | 0.0375628 | NA | 0.654676 | 1 | 48695658 | 0.0175187 | 32072 | 0.0320199 | 48695658 | 1.80E-14 | 1 | 389404 | 0.0019017 |
| 28 | rs11243143 | A | G | A | G | -0.035999 | 0.0156832 | NA | 0.532418 | 6 | 7120457 | 0.0175461 | 32072 | 0.371414 | 7120457 | 1.98E-84 | 6 | 389404 | 0.001849 |
| 29 | rs113392940 | T | G | T | G | 0.0162817 | -0.040377 | NA | 0.246615 | 19 | 18212786 | 0.0196858 | 32072 | 0.0402597 | 18212786 | 1.62E-15 | 19 | 389404 | 0.0020435 |
| 30 | rs113962566 | A | G | A | G | -0.269572 | -0.102829 | NA | 0.0180988 | 4 | 10185909 | 0.0662216 | 32072 | 0.120471 | 10185909 | 1.00E-200 | 4 | 389404 | 0.0071077 |
| 31 | rs114165349 | C | G | C | G | 0.0812338 | 0.0709248 | NA | 0.0181488 | 1 | 27021913 | 0.0607334 | 32072 | 0.242885 | 27021913 | 9.00E-43 | 1 | 389404 | 0.0059257 |
| 32 | rs114585931 | A | G | A | G | 0.112589 | -0.244044 | NA | 0.008872 | 4 | 10305155 | 0.106609 | 32072 | 0.0220709 | 10305155 | 1.87E-36 | 4 | 389404 | 0.0089289 |
| 33 | rs1150079 | T | A | T | A | 0.0103633 | -0.002684 | NA | 0.501464 | 6 | 111471425 | 0.0185453 | 32072 | 0.884921 | 111471425 | 7.95E-09 | 6 | 389404 | 0.0017962 |
| 34 | rs11556924 | T | C | T | C | -0.01064 | 0.0525956 | NA | 0.368403 | 7 | 129663496 | 0.0181365 | 32072 | 0.0037317 | 129663496 | 5.37E-09 | 7 | 389404 | 0.0018235 |
| 35 | rs11564722 | T | C | T | C | -0.024731 | -0.006127 | NA | 0.320332 | 11 | 2178330 | 0.0190116 | 32072 | 0.747228 | 2178330 | 2.13E-31 | 11 | 389404 | 0.0021217 |
| 36 | rs11587918 | A | T | A | T | 0.0119849 | 0.0236846 | NA | 0.199583 | 1 | 46161336 | 0.0203354 | 32072 | 0.244141 | 46161336 | 1.02E-08 | 1 | 389404 | 0.0020928 |
| 37 | rs11614136 | T | A | T | A | 0.0123093 | 0.0157929 | NA | 0.402088 | 12 | 52258777 | 0.0177453 | 32072 | 0.373479 | 52258777 | 3.51E-11 | 12 | 389404 | 0.0018585 |
| 38 | rs11649114 | G | A | G | A | 0.0289032 | 0.0159976 | NA | 0.151033 | 16 | 79924857 | 0.0244783 | 32072 | 0.513407 | 79924857 | 6.47E-31 | 16 | 389404 | 0.0025 |
| 39 | rs1165215 | A | G | A | G | 0.0671438 | -0.013217 | NA | 0.581319 | 6 | 25798932 | 0.0169808 | 32072 | 0.436373 | 25798932 | 1.00E-200 | 6 | 389404 | 0.0017959 |
| 40 | rs11657044 | C | T | C | T | 0.0229384 | 0.0020098 | NA | 0.777564 | 17 | 59450105 | 0.0217289 | 32072 | 0.926307 | 59450105 | 1.61E-21 | 17 | 389404 | 0.0024076 |
| 41 | rs11693363 | C | A | C | A | -0.032352 | 0.004127 | NA | 0.104969 | 2 | 69821008 | 0.027326 | 32072 | 0.879954 | 69821008 | 1.04E-34 | 2 | 389404 | 0.0026327 |
| 42 | rs117076370 | T | C | T | C | -0.030109 | 0.0658708 | NA | 0.0279765 | 9 | 95832927 | 0.0503515 | 32072 | 0.190799 | 95832927 | 2.32E-10 | 9 | 389404 | 0.0047503 |
| 43 | rs1171614 | C | T | C | T | 0.0580679 | -0.032025 | NA | 0.804543 | 10 | 61469538 | 0.0202861 | 32072 | 0.114408 | 61469538 | 4.81E-166 | 10 | 389404 | 0.0021144 |
| 44 | rs11735883 | A | G | A | G | -0.095215 | 0.0319326 | NA | 0.164065 | 4 | 9815762 | 0.0228004 | 32072 | 0.161356 | 9815762 | 1.00E-200 | 4 | 389404 | 0.0024774 |
| 45 | rs11757670 | T | C | T | C | -0.013529 | 0.0293274 | NA | 0.135465 | 6 | 105869130 | 0.0240929 | 32072 | 0.223503 | 105869130 | 4.91E-08 | 6 | 389404 | 0.0024803 |
| 46 | rs11768336 | T | C | T | C | 0.0132357 | 0.0069832 | NA | 0.354591 | 7 | 25791691 | 0.0187265 | 32072 | 0.70922 | 25791691 | 1.30E-10 | 7 | 389404 | 0.0020593 |
| 47 | rs118168183 | A | G | A | G | -0.031355 | -0.020947 | NA | 0.0212796 | 7 | 99291144 | 0.0560187 | 32072 | 0.708461 | 99291144 | 1.19E-09 | 7 | 389404 | 0.0051554 |
| 48 | rs11835818 | C | T | C | T | -0.018288 | 0.0042137 | NA | 0.497279 | 12 | 122494809 | 0.0171528 | 32072 | 0.805949 | 122494809 | 1.03E-24 | 12 | 389404 | 0.0017819 |
| 49 | rs11854957 | T | C | T | C | -0.01291 | 0.0300318 | NA | 0.1742 | 15 | 74467796 | 0.0217993 | 32072 | 0.168312 | 74467796 | 1.80E-09 | 15 | 389404 | 0.0021463 |
| 50 | rs11865952 | G | A | G | A | 0.0152977 | -0.001981 | NA | 0.437679 | 16 | 21092051 | 0.0171855 | 32072 | 0.908214 | 21092051 | 2.04E-17 | 16 | 389404 | 0.0018015 |
| 51 | rs1186699 | A | C | A | C | -0.013565 | 0.0080053 | NA | 0.353668 | 2 | 61662555 | 0.0176273 | 32072 | 0.649726 | 61662555 | 3.57E-13 | 2 | 389404 | 0.0018656 |
| 52 | rs11940694 | G | A | G | A | 0.0107649 | -0.021438 | NA | 0.549137 | 4 | 39414993 | 0.0171651 | 32072 | 0.211682 | 39414993 | 1.64E-08 | 4 | 389404 | 0.0019064 |
| 53 | rs12133907 | A | C | A | C | 0.0193806 | -0.029724 | NA | 0.614003 | 1 | 93850283 | 0.0176135 | 32072 | 0.0914892 | 93850283 | 1.56E-26 | 1 | 389404 | 0.001818 |
| 54 | rs12277177 | G | A | G | A | 0.0138947 | -0.033843 | NA | 0.152307 | 11 | 2926135 | 0.0221835 | 32072 | 0.127111 | 2926135 | 2.99E-09 | 11 | 389404 | 0.0023422 |
| 55 | rs1229984 | C | T | C | T | -0.057094 | 0.0019077 | NA | 0.82244 | 4 | 100239319 | 0.0323506 | 32072 | 0.952976 | 100239319 | 5.70E-20 | 4 | 389404 | 0.0062398 |
| 56 | rs12338812 | G | A | G | A | -0.018967 | 0.0251022 | NA | 0.119006 | 9 | 108536712 | 0.0325398 | 32072 | 0.440451 | 108536712 | 4.93E-08 | 9 | 389404 | 0.0034779 |
| 57 | rs12510175 | G | C | G | C | -0.014208 | -0.025266 | NA | 0.414515 | 4 | 146831323 | 0.0179766 | 32072 | 0.159873 | 146831323 | 3.24E-13 | 4 | 389404 | 0.0019506 |
| 58 | rs12542030 | G | C | G | C | -0.010657 | 5.57E-05 | NA | 0.607924 | 8 | 9985484 | 0.0177831 | 32072 | 0.997502 | 9985484 | 6.74E-09 | 8 | 389404 | 0.0018382 |
| 59 | rs12554192 | G | A | G | A | 0.0126782 | -0.044676 | NA | 0.257972 | 9 | 33084732 | 0.0188167 | 32072 | 0.0175837 | 33084732 | 2.34E-10 | 9 | 389404 | 0.0020007 |
| 60 | rs12576996 | G | T | G | T | 0.0387631 | -0.006842 | NA | 0.253333 | 11 | 65580638 | 0.0189521 | 32072 | 0.718098 | 65580638 | 2.60E-77 | 11 | 389404 | 0.0020828 |
| 61 | rs12593988 | G | A | G | A | 0.0125962 | 0.0177256 | NA | 0.824731 | 15 | 80705827 | 0.0211532 | 32072 | 0.402052 | 80705827 | 2.86E-08 | 15 | 389404 | 0.0022697 |
| 62 | rs1260326 | C | T | C | T | -0.047143 | 0.0336799 | NA | 0.565036 | 2 | 27730940 | 0.0174566 | 32072 | 0.0536883 | 27730940 | 2.86E-147 | 2 | 389404 | 0.0018242 |
| 63 | rs12708477 | C | A | C | A | -0.013256 | 0.0147469 | NA | 0.780812 | 15 | 63605954 | 0.0199482 | 32072 | 0.459749 | 63605954 | 4.26E-10 | 15 | 389404 | 0.002123 |
| 64 | rs12806743 | T | G | T | G | -0.01509 | -0.000653 | NA | 0.218525 | 11 | 30437981 | 0.0203181 | 32072 | 0.974352 | 30437981 | 1.03E-12 | 11 | 389404 | 0.0021175 |
| 65 | rs12911430 | T | A | T | A | 0.0112127 | 0.0117576 | NA | 0.383881 | 15 | 86011264 | 0.0170232 | 32072 | 0.489769 | 86011264 | 4.66E-10 | 15 | 389404 | 0.0017998 |
| 66 | rs13015552 | T | C | T | C | 0.017395 | 0.0165312 | NA | 0.409741 | 2 | 148843372 | 0.0172531 | 32072 | 0.337985 | 148843372 | 2.79E-21 | 2 | 389404 | 0.0018368 |
| 67 | rs13107325 | T | C | T | C | -0.025631 | 0.025279 | NA | 0.0674602 | 4 | 103188709 | 0.0317715 | 32072 | 0.426237 | 103188709 | 3.25E-13 | 4 | 389404 | 0.0035191 |
| 68 | rs13117384 | T | C | T | C | 0.0110153 | -0.032621 | NA | 0.478174 | 4 | 128069140 | 0.0171325 | 32072 | 0.0569049 | 128069140 | 2.92E-09 | 4 | 389404 | 0.0018557 |
| 69 | rs1317983 | C | T | C | T | 0.0326501 | 0.0164725 | NA | 0.718575 | 6 | 43806335 | 0.0185118 | 32072 | 0.373554 | 43806335 | 2.69E-64 | 6 | 389404 | 0.0019285 |
| 70 | rs13234378 | T | A | T | A | -0.042789 | -0.005912 | NA | 0.119679 | 7 | 73026151 | 0.0260198 | 32072 | 0.820245 | 73026151 | 1.48E-58 | 7 | 389404 | 0.0026522 |
| 71 | rs13316 | A | C | A | C | -0.011474 | 0.028372 | NA | 0.432491 | 14 | 93407301 | 0.0174794 | 32072 | 0.104553 | 93407301 | 3.06E-10 | 14 | 389404 | 0.0018224 |
| 72 | rs13411042 | A | C | A | C | 0.0151629 | -0.047793 | NA | 0.494956 | 2 | 183087067 | 0.0171026 | 32072 | 0.0051988 | 183087067 | 1.94E-17 | 2 | 389404 | 0.0017845 |
| 73 | rs1407555 | C | T | C | T | -0.011897 | 2.81E-05 | NA | 0.480832 | 1 | 185122022 | 0.017216 | 32072 | 0.998695 | 185122022 | 2.61E-11 | 1 | 389404 | 0.0017844 |
| 74 | rs1420473 | T | A | T | A | 0.012696 | -0.002047 | NA | 0.485735 | 3 | 168782038 | 0.0169869 | 32072 | 0.90409 | 168782038 | 1.47E-12 | 3 | 389404 | 0.0017939 |
| 75 | rs1446585 | G | A | G | A | -0.011801 | 0.0844749 | NA | 0.526987 | 2 | 136407479 | 0.0209071 | 32072 | 5.33E-05 | 136407479 | 3.28E-08 | 2 | 389404 | 0.0021357 |
| 76 | rs145148878 | T | C | T | C | 0.0358081 | -0.033183 | NA | 0.014091 | 5 | 137494358 | 0.0738575 | 32072 | 0.653224 | 137494358 | 4.67E-08 | 5 | 389404 | 0.006554 |
| 77 | rs1454687 | G | C | G | C | -0.011055 | 0.0062586 | NA | 0.538097 | 3 | 94038085 | 0.0170098 | 32072 | 0.712919 | 94038085 | 5.16E-10 | 3 | 389404 | 0.0017789 |
| 78 | rs146668947 | T | A | T | A | -0.091843 | -0.022038 | NA | 0.0104169 | 4 | 8764853 | 0.141245 | 32072 | 0.876014 | 8764853 | 8.63E-16 | 4 | 389404 | 0.0114164 |
| 79 | rs146787580 | C | A | C | A | 0.0273839 | -0.004702 | NA | 0.106667 | 2 | 203431804 | 0.0272017 | 32072 | 0.862772 | 203412513 | 3.67E-22 | 2 | 389404 | 0.0028289 |
| 80 | rs148633069 | G | T | G | T | -0.243862 | 0.0122319 | NA | 0.0128217 | 4 | 10085102 | 0.0689429 | 32072 | 0.859178 | 10085102 | 1.85E-171 | 4 | 389404 | 0.0087365 |
| 81 | rs150147865 | T | A | T | A | -0.100165 | -0.054939 | NA | 0.0116025 | 1 | 120326253 | 0.0902956 | 32072 | 0.542898 | 120326253 | 9.46E-23 | 1 | 389404 | 0.0102027 |
| 82 | rs151277284 | A | G | A | G | -0.259124 | -0.030227 | NA | 0.0150419 | 4 | 9655570 | 0.139024 | 32072 | 0.827878 | 9655570 | 1.00E-200 | 4 | 389404 | 0.0083627 |
| 83 | rs1551210 | T | C | T | C | -0.011971 | -0.008493 | NA | 0.389277 | 12 | 42865874 | 0.0176494 | 32072 | 0.630376 | 42865874 | 3.13E-11 | 12 | 389404 | 0.0018028 |
| 84 | rs1684662 | A | T | A | T | -0.011804 | -0.011502 | NA | 0.740527 | 17 | 42180822 | 0.0191505 | 32072 | 0.548104 | 42180822 | 6.72E-09 | 17 | 389404 | 0.002036 |
| 85 | rs16942751 | A | C | A | C | 0.0185725 | -0.017718 | NA | 0.136208 | 18 | 24393213 | 0.0266251 | 32072 | 0.505757 | 24393213 | 5.13E-09 | 18 | 389404 | 0.0031787 |
| 86 | rs17020588 | A | C | A | C | -0.016363 | 0.0352107 | NA | 0.224923 | 4 | 146720661 | 0.0240638 | 32072 | 0.143407 | 146720661 | 8.73E-10 | 4 | 389404 | 0.0026688 |
| 87 | rs17050272 | A | G | A | G | 0.0242134 | 0.016833 | NA | 0.418572 | 2 | 121306440 | 0.0173439 | 32072 | 0.331776 | 121306440 | 9.26E-41 | 2 | 389404 | 0.0018113 |
| 88 | rs1705614 | T | C | T | C | 0.0233002 | 0.0062987 | NA | 0.737292 | 3 | 141811257 | 0.0194739 | 32072 | 0.746361 | 141811257 | 1.39E-31 | 3 | 389404 | 0.0019927 |
| 89 | rs1713815 | C | T | C | T | 0.0187059 | -0.009472 | NA | 0.873083 | 3 | 153872445 | 0.0257637 | 32072 | 0.713137 | 153872445 | 2.31E-14 | 3 | 389404 | 0.0024509 |
| 90 | rs17300741 | G | A | G | A | -0.054716 | -0.000997 | NA | 0.461351 | 11 | 64331462 | 0.0170133 | 32072 | 0.953265 | 64331462 | 1.00E-200 | 11 | 389404 | 0.0017954 |
| 91 | rs174603 | A | G | A | G | 0.0134985 | -0.039518 | NA | 0.276617 | 11 | 61626206 | 0.0186755 | 32072 | 0.0343439 | 61626206 | 2.27E-12 | 11 | 389404 | 0.0019237 |
| 92 | rs17473100 | C | T | C | T | 0.0120377 | 0.0212207 | NA | 0.263623 | 12 | 23861993 | 0.019046 | 32072 | 0.265202 | 23861993 | 1.74E-09 | 12 | 389404 | 0.0019994 |
| 93 | rs17592117 | C | T | C | T | 0.0426326 | -0.089115 | NA | 0.0933188 | 10 | 52643038 | 0.0272586 | 32072 | 0.0010784 | 52643038 | 1.34E-55 | 10 | 389404 | 0.0027141 |
| 94 | rs17632159 | C | G | C | G | -0.034492 | -0.013909 | NA | 0.292685 | 5 | 72431482 | 0.0187926 | 32072 | 0.459238 | 72431482 | 4.90E-71 | 5 | 389404 | 0.0019355 |
| 95 | rs1800961 | T | C | T | C | -0.049691 | -0.054688 | NA | 0.027395 | 20 | 43042364 | 0.0483095 | 32072 | 0.25762 | 43042364 | 1.54E-22 | 20 | 389404 | 0.0050871 |
| 96 | rs181673 | C | A | C | A | 0.0162981 | -0.017559 | NA | 0.605307 | 10 | 119388528 | 0.0172767 | 32072 | 0.309465 | 119388528 | 5.62E-20 | 10 | 389404 | 0.0017809 |
| 97 | rs1848968 | T | C | T | C | -0.013669 | -0.003243 | NA | 0.307887 | 12 | 78794166 | 0.0179844 | 32072 | 0.856895 | 78794166 | 1.71E-13 | 12 | 389404 | 0.0018547 |
| 98 | rs1851285 | G | C | G | C | 0.0139934 | -0.007784 | NA | 0.537132 | 2 | 213125286 | 0.0171871 | 32072 | 0.650616 | 213125286 | 6.64E-15 | 2 | 389404 | 0.0017961 |
| 99 | rs187355703 | G | C | G | C | 0.0673201 | -0.071465 | NA | 0.0166351 | 2 | 176993583 | 0.0626515 | 32072 | 0.254006 | 176993583 | 2.00E-32 | 2 | 389404 | 0.0056781 |
| 100 | rs1887249 | C | T | C | T | 0.0135702 | -0.005078 | NA | 0.641823 | 1 | 82954121 | 0.0181237 | 32072 | 0.779327 | 82954121 | 2.68E-13 | 1 | 389404 | 0.0018565 |
| 101 | rs189396107 | A | G | A | G | 0.0224724 | 0.0090175 | NA | 0.0509334 | 6 | 55607900 | 0.0361278 | 32072 | 0.802897 | 55601428 | 1.93E-10 | 6 | 389404 | 0.0035297 |
| 102 | rs1899951 | T | C | T | C | -0.018356 | -0.014351 | NA | 0.108241 | 3 | 12394840 | 0.0264691 | 32072 | 0.587704 | 12394840 | 1.39E-11 | 3 | 389404 | 0.0027156 |
| 103 | rs1929926 | T | C | T | C | -0.011952 | 0.0111212 | NA | 0.297163 | 6 | 42744070 | 0.0197738 | 32072 | 0.573828 | 42744070 | 4.26E-10 | 6 | 389404 | 0.001914 |
| 104 | rs193220 | T | C | T | C | -0.015773 | 0.0233211 | NA | 0.367066 | 17 | 74268619 | 0.0182073 | 32072 | 0.20024 | 74268619 | 1.53E-16 | 17 | 389404 | 0.0019109 |
| 105 | rs1940804 | T | C | T | C | -0.013838 | 0.0128393 | NA | 0.634814 | 11 | 119237217 | 0.0174532 | 32072 | 0.461949 | 119237217 | 6.24E-14 | 11 | 389404 | 0.0018443 |
| 106 | rs1965132 | A | C | A | C | 0.0136056 | -0.01625 | NA | 0.50283 | 3 | 69147519 | 0.0170185 | 32072 | 0.339649 | 69147519 | 3.46E-14 | 3 | 389404 | 0.001795 |
| 107 | rs2132276 | A | G | A | G | 0.0139821 | 0.0224369 | NA | 0.492005 | 7 | 97826340 | 0.0170881 | 32072 | 0.189178 | 97826340 | 4.04E-15 | 7 | 389404 | 0.0017803 |
| 108 | rs2137683 | G | C | G | C | -0.032938 | -0.029784 | NA | 0.603669 | 15 | 99289638 | 0.0176591 | 32072 | 0.0916811 | 99289638 | 1.39E-70 | 15 | 389404 | 0.0018544 |
| 109 | rs2168711 | C | T | C | T | 0.0202121 | 0.0201499 | NA | 0.237004 | 18 | 57848531 | 0.0200324 | 32072 | 0.314481 | 57848531 | 5.24E-22 | 18 | 389404 | 0.0020959 |
| 110 | rs219778 | G | A | G | A | -0.022473 | -0.028634 | NA | 0.24188 | 21 | 37834641 | 0.0200806 | 32072 | 0.153877 | 37834641 | 5.12E-29 | 21 | 389404 | 0.0020102 |
| 111 | rs2226399 | C | T | C | T | -0.011634 | 0.0102206 | NA | 0.501916 | 8 | 102654182 | 0.0169638 | 32072 | 0.546845 | 102654182 | 7.76E-11 | 8 | 389404 | 0.0017884 |
| 112 | rs2229357 | A | G | A | G | -0.048659 | 0.0147946 | NA | 0.199755 | 12 | 57843711 | 0.0207929 | 32072 | 0.476762 | 57843711 | 1.05E-122 | 12 | 389404 | 0.0020656 |
| 113 | rs2258043 | C | T | C | T | -0.013434 | 0.0378863 | NA | 0.525982 | 12 | 121451425 | 0.0175019 | 32072 | 0.0304103 | 121451425 | 4.41E-14 | 12 | 389404 | 0.0017798 |
| 114 | rs2279620 | C | G | C | G | 0.0191412 | -0.032032 | NA | 0.136786 | 17 | 7792465 | 0.0286895 | 32072 | 0.264199 | 7792465 | 9.63E-12 | 17 | 389404 | 0.00281 |
| 115 | rs2305278 | G | C | G | C | 0.0156673 | 0.0328642 | NA | 0.209329 | 1 | 205028581 | 0.022204 | 32072 | 0.138846 | 205028581 | 1.21E-11 | 1 | 389404 | 0.0023112 |
| 116 | rs2322603 | C | T | C | T | -0.009838 | 0.0230953 | NA | 0.483256 | 8 | 27163123 | 0.0170275 | 32072 | 0.174987 | 27163123 | 4.01E-08 | 8 | 389404 | 0.0017918 |
| 117 | rs2345962 | G | A | G | A | 0.0155017 | 0.0089736 | NA | 0.514367 | 1 | 163741788 | 0.0174544 | 32072 | 0.60717 | 163741788 | 4.12E-18 | 1 | 389404 | 0.0017869 |
| 118 | rs234951 | A | G | A | G | -0.012683 | -0.004782 | NA | 0.665036 | 6 | 2201424 | 0.0184752 | 32072 | 0.795757 | 2201424 | 9.21E-12 | 6 | 389404 | 0.0018602 |
| 119 | rs2437700 | T | C | T | C | 0.0111155 | -0.016623 | NA | 0.304409 | 6 | 80194343 | 0.0184227 | 32072 | 0.366885 | 80188960 | 5.65E-09 | 6 | 389404 | 0.0019076 |
| 120 | rs2439823 | G | A | G | A | 0.0111461 | -0.003732 | NA | 0.500553 | 10 | 99778226 | 0.0172191 | 32072 | 0.828418 | 99778226 | 4.96E-10 | 10 | 389404 | 0.0017919 |
| 121 | rs2449451 | G | T | G | T | 0.0121393 | -0.006523 | NA | 0.64429 | 5 | 55447339 | 0.0183208 | 32072 | 0.721805 | 55447339 | 1.67E-10 | 5 | 389404 | 0.0019 |
| 122 | rs2470548 | A | G | A | G | -0.013009 | -0.032244 | NA | 0.584322 | 3 | 15737689 | 0.017534 | 32072 | 0.0659204 | 15737689 | 7.13E-13 | 3 | 389404 | 0.0018126 |
| 123 | rs2480712 | C | G | C | G | 0.0145137 | -0.037711 | NA | 0.663285 | 1 | 2156999 | 0.0176425 | 32072 | 0.0325582 | 2156999 | 1.82E-14 | 1 | 389404 | 0.0018941 |
| 124 | rs2493121 | A | T | A | T | 0.0121012 | 0.0193294 | NA | 0.693839 | 1 | 179272116 | 0.0178894 | 32072 | 0.279923 | 179272116 | 8.75E-11 | 1 | 389404 | 0.0018654 |
| 125 | rs2540034 | T | C | T | C | 0.0112774 | -0.010436 | NA | 0.524891 | 16 | 4022694 | 0.0174022 | 32072 | 0.548713 | 4022694 | 6.41E-10 | 16 | 389404 | 0.0018248 |
| 126 | rs255751 | T | C | T | C | 0.0193603 | -0.008803 | NA | 0.703521 | 5 | 53319517 | 0.018161 | 32072 | 0.627869 | 53319517 | 2.24E-23 | 5 | 389404 | 0.0019434 |
| 127 | rs2647172 | A | C | A | C | 0.0114423 | 0.0009609 | NA | 0.714473 | 1 | 17431681 | 0.0188766 | 32072 | 0.959402 | 17431681 | 7.67E-09 | 1 | 389404 | 0.0019812 |
| 128 | rs2683123 | C | T | C | T | 0.0108628 | 0.0009464 | NA | 0.524648 | 15 | 36149397 | 0.0173368 | 32072 | 0.956465 | 36124213 | 2.28E-09 | 15 | 389404 | 0.0018177 |
| 129 | rs2695571 | A | G | A | G | 0.0102652 | 0.0248333 | NA | 0.392518 | 7 | 156391043 | 0.0176478 | 32072 | 0.15938 | 156389139 | 2.62E-08 | 7 | 389404 | 0.0018446 |
| 130 | rs272889 | G | A | G | A | 0.0149021 | 0.0200631 | NA | 0.57867 | 5 | 131665378 | 0.0174447 | 32072 | 0.250105 | 131665378 | 6.03E-16 | 5 | 389404 | 0.0018423 |
| 131 | rs2788146 | C | T | C | T | 0.0356598 | -0.006938 | NA | 0.028727 | 1 | 212070975 | 0.0484517 | 32072 | 0.886137 | 212070975 | 5.64E-14 | 1 | 389404 | 0.0047444 |
| 132 | rs2823139 | A | G | A | G | 0.0153572 | 0.0181909 | NA | 0.314145 | 21 | 16576783 | 0.018143 | 32072 | 0.316033 | 16576783 | 3.77E-16 | 21 | 389404 | 0.0018853 |
| 133 | rs2834317 | A | G | A | G | 0.0171509 | -0.006343 | NA | 0.134273 | 21 | 35356706 | 0.0244617 | 32072 | 0.795404 | 35356706 | 4.43E-12 | 21 | 389404 | 0.0024775 |
| 134 | rs28517717 | T | C | T | C | -0.022807 | -0.014295 | NA | 0.319076 | 9 | 33179451 | 0.0191619 | 32072 | 0.45567 | 33179451 | 7.04E-30 | 9 | 389404 | 0.0020086 |
| 135 | rs28621809 | T | C | T | C | -0.011447 | 0.0079143 | NA | 0.430131 | 15 | 51101606 | 0.0172638 | 32072 | 0.646644 | 51101606 | 1.88E-10 | 15 | 389404 | 0.0017968 |
| 136 | rs28634790 | A | G | A | G | 0.117509 | -0.062181 | NA | 0.111091 | 4 | 88954523 | 0.0301594 | 32072 | 0.0392338 | 88954523 | 1.00E-200 | 4 | 389404 | 0.0030861 |
| 137 | rs2941471 | A | G | A | G | 0.0320743 | 0.0283735 | NA | 0.552235 | 8 | 76470404 | 0.0171274 | 32072 | 0.0975955 | 76470404 | 1.24E-71 | 8 | 389404 | 0.0017922 |
| 138 | rs2943654 | T | C | T | C | 0.0125138 | 0.0250138 | NA | 0.688891 | 2 | 227112754 | 0.0177728 | 32072 | 0.159302 | 227112754 | 1.81E-11 | 2 | 389404 | 0.001862 |
| 139 | rs2950790 | A | G | A | G | -0.012277 | -0.027642 | NA | 0.769546 | 2 | 145759721 | 0.0195819 | 32072 | 0.15807 | 145759721 | 2.46E-09 | 2 | 389404 | 0.0020585 |
| 140 | rs316012 | C | T | C | T | -0.012263 | 0.0214172 | NA | 0.772604 | 6 | 160673884 | 0.0199684 | 32072 | 0.28347 | 160673884 | 2.75E-09 | 6 | 389404 | 0.0020624 |
| 141 | rs34338229 | A | G | A | G | 0.0105929 | 0.0017291 | NA | 0.360687 | 6 | 15370313 | 0.0178203 | 32072 | 0.922704 | 15370313 | 2.38E-08 | 6 | 389404 | 0.0018978 |
| 142 | rs343907 | A | G | A | G | 0.0137058 | 0.0149745 | NA | 0.696215 | 15 | 33339807 | 0.0193613 | 32072 | 0.43927 | 33339807 | 2.11E-11 | 15 | 389404 | 0.0020462 |
| 143 | rs34811474 | A | G | A | G | -0.012534 | -0.023968 | NA | 0.191584 | 4 | 25408838 | 0.0227649 | 32072 | 0.292402 | 25408838 | 9.65E-09 | 4 | 389404 | 0.0021849 |
| 144 | rs35399441 | C | A | C | A | 0.0132571 | 0.003868 | NA | 0.198262 | 8 | 57089431 | 0.0206859 | 32072 | 0.851673 | 57089431 | 5.06E-09 | 8 | 389404 | 0.0022681 |
| 145 | rs358226 | G | A | G | A | 0.0172563 | 0.0302409 | NA | 0.828886 | 4 | 22825055 | 0.0237423 | 32072 | 0.202765 | 22825055 | 3.89E-11 | 4 | 389404 | 0.0026113 |
| 146 | rs36025723 | T | C | T | C | -0.015094 | 0.0063012 | NA | 0.114348 | 1 | 235106914 | 0.0253474 | 32072 | 0.803677 | 235106914 | 1.17E-08 | 1 | 389404 | 0.0026463 |
| 147 | rs3746574 | C | T | C | T | -0.01327 | -0.034235 | NA | 0.51492 | 20 | 43058018 | 0.0177035 | 32072 | 0.0531374 | 43058018 | 2.40E-13 | 20 | 389404 | 0.0018118 |
| 148 | rs375303233 | T | G | T | G | -0.014554 | -0.002037 | NA | 0.305987 | 5 | 90243899 | 0.0186742 | 32072 | 0.913142 | 90222937 | 3.66E-13 | 5 | 389404 | 0.0020026 |
| 149 | rs3786900 | G | A | G | A | -0.012982 | 0.0086634 | NA | 0.274807 | 19 | 33897149 | 0.0193715 | 32072 | 0.654715 | 33897149 | 1.02E-10 | 19 | 389404 | 0.0020086 |
| 150 | rs3789061 | C | T | C | T | -0.015404 | -0.025059 | NA | 0.440077 | 2 | 111918230 | 0.0172365 | 32072 | 0.145995 | 111918230 | 6.68E-18 | 2 | 389404 | 0.001787 |
| 151 | rs3794748 | G | A | G | A | -0.030865 | -0.030567 | NA | 0.608401 | 17 | 53365172 | 0.0176579 | 32072 | 0.0834373 | 53365172 | 1.43E-63 | 17 | 389404 | 0.0018338 |
| 152 | rs3822858 | C | T | C | T | -0.013016 | 0.0005606 | NA | 0.34279 | 6 | 116305064 | 0.0176947 | 32072 | 0.974726 | 116305064 | 7.04E-13 | 6 | 389404 | 0.0018132 |
| 153 | rs3824359 | C | T | C | T | 0.0160499 | -0.006688 | NA | 0.166095 | 9 | 139105229 | 0.0238475 | 32072 | 0.779123 | 139105229 | 2.74E-10 | 9 | 389404 | 0.0025423 |
| 154 | rs3902354 | A | C | A | C | -0.011123 | 0.0050236 | NA | 0.709738 | 1 | 109819296 | 0.0179838 | 32072 | 0.779983 | 109819296 | 4.86E-09 | 1 | 389404 | 0.0019008 |
| 155 | rs40270 | C | A | C | A | 0.0206154 | 0.0332007 | NA | 0.68077 | 5 | 55804552 | 0.0200966 | 32072 | 0.0985213 | 55804552 | 2.20E-22 | 5 | 389404 | 0.0021183 |
| 156 | rs4140872 | T | C | T | C | -0.028292 | 0.0020222 | NA | 0.713065 | 2 | 170026596 | 0.0198487 | 32072 | 0.918851 | 170026596 | 2.82E-42 | 2 | 389404 | 0.0020764 |
| 157 | rs4149056 | C | T | C | T | -0.017973 | -0.007478 | NA | 0.163511 | 12 | 21331549 | 0.0226483 | 32072 | 0.741262 | 21331549 | 4.95E-13 | 12 | 389404 | 0.002487 |
| 158 | rs4244340 | C | G | C | G | -0.01274 | -0.024461 | NA | 0.828194 | 10 | 102499268 | 0.0211537 | 32072 | 0.247547 | 102499268 | 8.52E-09 | 10 | 389404 | 0.0022126 |
| 159 | rs4285082 | T | C | T | C | -0.070533 | -0.092419 | NA | 0.980076 | 4 | 10802922 | 0.0594119 | 32072 | 0.119811 | 10802922 | 2.20E-27 | 4 | 389404 | 0.0065061 |
| 160 | rs4415952 | C | T | C | T | 0.014028 | -0.009297 | NA | 0.712849 | 14 | 73189153 | 0.0184264 | 32072 | 0.613889 | 73189153 | 2.87E-12 | 14 | 389404 | 0.0020087 |
| 161 | rs4468717 | T | C | T | C | -0.023547 | -0.088613 | NA | 0.0735041 | 18 | 3457606 | 0.0366182 | 32072 | 0.0155246 | 3457606 | 1.21E-12 | 18 | 389404 | 0.0033145 |
| 162 | rs4491726 | G | A | G | A | 0.0190237 | -0.015091 | NA | 0.279967 | 2 | 18676276 | 0.0182013 | 32072 | 0.407044 | 18676276 | 2.83E-23 | 2 | 389404 | 0.0019141 |
| 163 | rs455213 | C | T | C | T | 0.01956 | 9.77E-05 | NA | 0.42941 | 5 | 34660235 | 0.0171955 | 32072 | 0.995469 | 34660235 | 6.28E-28 | 5 | 389404 | 0.0017855 |
| 164 | rs45619139 | G | C | G | C | 0.0188335 | -0.034921 | NA | 0.092364 | 10 | 16940846 | 0.0272269 | 32072 | 0.199635 | 16940846 | 1.83E-10 | 10 | 389404 | 0.0029542 |
| 165 | rs4575545 | A | G | A | G | -0.026875 | 0.0054874 | NA | 0.341184 | 16 | 79755446 | 0.0187737 | 32072 | 0.770064 | 79755446 | 7.20E-44 | 16 | 389404 | 0.0019347 |
| 166 | rs4675812 | A | G | A | G | -0.016766 | -0.021735 | NA | 0.600449 | 2 | 242395674 | 0.0172145 | 32072 | 0.206737 | 242395674 | 1.88E-20 | 2 | 389404 | 0.0018088 |
| 167 | rs4687694 | C | T | C | T | 0.0348832 | -0.047627 | NA | 0.440642 | 3 | 53049047 | 0.0175961 | 32072 | 0.0067964 | 53049047 | 1.03E-83 | 3 | 389404 | 0.0017995 |
| 168 | rs4744712 | C | A | C | A | 0.011733 | 0.0278443 | NA | 0.603128 | 9 | 71434707 | 0.0172968 | 32072 | 0.107443 | 71434707 | 1.02E-10 | 9 | 389404 | 0.001815 |
| 169 | rs4751640 | C | A | C | A | -0.014562 | 0.001132 | NA | 0.67582 | 10 | 119572168 | 0.0183031 | 32072 | 0.950686 | 119572168 | 7.63E-14 | 10 | 389404 | 0.0019477 |
| 170 | rs4752992 | T | G | T | G | -0.015606 | -0.011985 | NA | 0.167246 | 11 | 47410751 | 0.0252286 | 32072 | 0.634736 | 47410751 | 9.16E-10 | 11 | 389404 | 0.0025485 |
| 171 | rs478425 | T | G | T | G | -0.014053 | 0.0417907 | NA | 0.396413 | 1 | 234863602 | 0.0175495 | 32072 | 0.0172516 | 234863602 | 2.84E-14 | 1 | 389404 | 0.0018478 |
| 172 | rs4823085 | C | T | C | T | 0.0144963 | 0.030911 | NA | 0.841896 | 22 | 30686226 | 0.0225692 | 32072 | 0.170808 | 30686226 | 5.23E-11 | 22 | 389404 | 0.0022084 |
| 173 | rs4844981 | C | T | C | T | -0.011979 | 0.0206067 | NA | 0.370349 | 1 | 210397156 | 0.0184559 | 32072 | 0.264192 | 210397156 | 1.20E-09 | 1 | 389404 | 0.0019699 |
| 174 | rs4884958 | A | G | A | G | -0.017149 | -0.020518 | NA | 0.466971 | 13 | 72346092 | 0.017735 | 32072 | 0.247309 | 72345448 | 8.91E-21 | 13 | 389404 | 0.0018345 |
| 175 | rs4938640 | C | G | C | G | -0.014831 | 0.0121133 | NA | 0.884628 | 11 | 111323581 | 0.0270788 | 32072 | 0.654633 | 111323581 | 3.32E-08 | 11 | 389404 | 0.0026849 |
| 176 | rs508205 | A | G | A | G | 0.0143023 | -0.018918 | NA | 0.581481 | 11 | 120057343 | 0.0172148 | 32072 | 0.271787 | 120057343 | 1.56E-15 | 11 | 389404 | 0.0017941 |
| 177 | rs541564 | A | G | A | G | 0.0147449 | -0.012841 | NA | 0.417017 | 10 | 69848298 | 0.0181978 | 32072 | 0.480401 | 69848298 | 2.97E-15 | 10 | 389404 | 0.0018683 |
| 178 | rs55838345 | C | G | C | G | 0.018247 | -0.027978 | NA | 0.105106 | 10 | 82085120 | 0.0261749 | 32072 | 0.285125 | 82085120 | 1.78E-11 | 10 | 389404 | 0.0027143 |
| 179 | rs55924362 | G | A | G | A | -0.031717 | -0.000111 | NA | 0.0875355 | 4 | 89075031 | 0.028906 | 32072 | 0.996932 | 89075031 | 7.76E-27 | 4 | 389404 | 0.0029573 |
| 180 | rs55935060 | C | G | C | G | -0.020412 | -0.011437 | NA | 0.139001 | 2 | 32756741 | 0.0234045 | 32072 | 0.625086 | 32756741 | 4.14E-15 | 2 | 389404 | 0.0026001 |
| 181 | rs56240036 | T | C | T | C | -0.016897 | -0.000655 | NA | 0.301981 | 7 | 101231082 | 0.0181368 | 32072 | 0.971185 | 101231082 | 5.64E-19 | 7 | 389404 | 0.0018987 |
| 182 | rs56292253 | A | G | A | G | 0.0818416 | -0.048999 | NA | 0.0187718 | 4 | 10263571 | 0.0622438 | 32072 | 0.431161 | 10263571 | 1.06E-46 | 4 | 389404 | 0.0057031 |
| 183 | rs56379622 | A | G | A | G | -0.033039 | 0.0493917 | NA | 0.0483518 | 9 | 130756222 | 0.0366153 | 32072 | 0.177358 | 130756222 | 1.53E-14 | 9 | 389404 | 0.0042992 |
| 184 | rs57158761 | G | A | G | A | 0.0121016 | 0.0026321 | NA | 0.44276 | 3 | 185371172 | 0.0173436 | 32072 | 0.879377 | 185371172 | 1.62E-11 | 3 | 389404 | 0.0017964 |
| 185 | rs59215427 | A | C | A | C | -0.01098 | -0.019425 | NA | 0.3933 | 4 | 101132753 | 0.0182659 | 32072 | 0.287577 | 101132753 | 1.26E-08 | 4 | 389404 | 0.0019293 |
| 186 | rs6026580 | T | C | T | C | -0.013384 | -0.007971 | NA | 0.67098 | 20 | 57469073 | 0.018026 | 32072 | 0.658347 | 57468150 | 1.12E-12 | 20 | 389404 | 0.0018813 |
| 187 | rs6040060 | G | A | G | A | -0.014161 | 0.0240143 | NA | 0.752777 | 20 | 10640201 | 0.0203224 | 32072 | 0.237337 | 10640201 | 2.52E-12 | 20 | 389404 | 0.0020223 |
| 188 | rs6127099 | T | A | T | A | -0.01641 | -0.014565 | NA | 0.306196 | 20 | 52731402 | 0.0186351 | 32072 | 0.434465 | 52731402 | 5.30E-16 | 20 | 389404 | 0.0020249 |
| 189 | rs61344778 | T | C | T | C | 0.0125956 | -0.015364 | NA | 0.262458 | 8 | 32276003 | 0.0183922 | 32072 | 0.403534 | 32268420 | 1.76E-10 | 8 | 389404 | 0.001974 |
| 190 | rs6142206 | A | G | A | G | 0.0156399 | -0.049977 | NA | 0.387851 | 20 | 33212055 | 0.0171569 | 32072 | 0.0035805 | 33212055 | 3.44E-18 | 20 | 389404 | 0.0017985 |
| 191 | rs61795284 | C | A | C | A | -0.039294 | 0.0822984 | NA | 0.0102745 | 4 | 11071065 | 0.0808261 | 32072 | 0.308575 | 11071065 | 3.19E-08 | 4 | 389404 | 0.007105 |
| 192 | rs62033399 | T | C | T | C | 0.018388 | -0.007268 | NA | 0.377027 | 16 | 53810943 | 0.0172073 | 32072 | 0.672729 | 53810943 | 5.37E-24 | 16 | 389404 | 0.0018201 |
| 193 | rs62078746 | A | G | A | G | -0.010456 | -0.030624 | NA | 0.457044 | 17 | 80053590 | 0.0175905 | 32072 | 0.081694 | 80053590 | 5.21E-09 | 17 | 389404 | 0.0017902 |
| 194 | rs62106258 | C | T | C | T | -0.033669 | -0.07713 | NA | 0.0414273 | 2 | 417167 | 0.0432784 | 32072 | 0.0747205 | 417167 | 4.91E-16 | 2 | 389404 | 0.0041496 |
| 195 | rs62262727 | A | G | A | G | 0.034203 | -0.006363 | NA | 0.0525195 | 3 | 49803395 | 0.0366283 | 32072 | 0.862096 | 49803395 | 1.51E-22 | 3 | 389404 | 0.0035008 |
| 196 | rs62279635 | A | G | A | G | 0.0122698 | 0.0089749 | NA | 0.296966 | 3 | 156985708 | 0.0197539 | 32072 | 0.649587 | 156985708 | 1.25E-09 | 3 | 389404 | 0.0020201 |
| 197 | rs62294340 | A | G | A | G | -0.017631 | -0.014566 | NA | 0.35183 | 3 | 169155476 | 0.0178748 | 32072 | 0.415153 | 169155476 | 5.84E-22 | 3 | 389404 | 0.0018304 |
| 198 | rs62397245 | G | C | G | C | 0.0191858 | 0.0044038 | NA | 0.210472 | 5 | 176750688 | 0.0199226 | 32072 | 0.825057 | 176750688 | 4.12E-19 | 5 | 389404 | 0.0021476 |
| 199 | rs62398607 | C | T | C | T | -0.015782 | 0.0022383 | NA | 0.206908 | 6 | 39145523 | 0.0206993 | 32072 | 0.91389 | 39145523 | 6.04E-13 | 6 | 389404 | 0.002192 |
| 200 | rs62435145 | T | G | T | G | 0.0341453 | -0.009943 | NA | 0.618224 | 7 | 1286567 | 0.018146 | 32072 | 0.583729 | 1286567 | 3.21E-66 | 7 | 389404 | 0.0019865 |
| 201 | rs62580766 | T | C | T | C | -0.013973 | 0.019787 | NA | 0.168529 | 9 | 113034490 | 0.0214736 | 32072 | 0.356812 | 113034490 | 1.25E-09 | 9 | 389404 | 0.0023005 |
| 202 | rs62618693 | T | C | T | C | -0.024707 | 0.111771 | NA | 0.0317316 | 11 | 32956492 | 0.0457771 | 32072 | 0.0146211 | 32956492 | 8.00E-09 | 11 | 389404 | 0.0042832 |
| 203 | rs6415788 | T | G | T | G | -0.011945 | 0.0001741 | NA | 0.644119 | 9 | 4118111 | 0.0175395 | 32072 | 0.992078 | 4118111 | 8.34E-11 | 9 | 389404 | 0.0018393 |
| 204 | rs6504974 | C | A | C | A | -0.012991 | 0.0191393 | NA | 0.417736 | 17 | 45674998 | 0.0172766 | 32072 | 0.267942 | 45597726 | 3.84E-13 | 17 | 389404 | 0.0017892 |
| 205 | rs653178 | T | C | T | C | -0.029027 | 0.0769157 | NA | 0.579198 | 12 | 112007756 | 0.0170929 | 32072 | 6.80E-06 | 112007756 | 8.15E-60 | 12 | 389404 | 0.0017795 |
| 206 | rs66485119 | A | C | A | C | -0.020081 | -0.040014 | NA | 0.86552 | 8 | 103652686 | 0.0257496 | 32072 | 0.120195 | 103652686 | 2.09E-15 | 8 | 389404 | 0.0025305 |
| 207 | rs6679229 | G | A | G | A | -0.021798 | -0.000364 | NA | 0.334621 | 1 | 15914078 | 0.0187523 | 32072 | 0.984495 | 15914078 | 4.90E-30 | 1 | 389404 | 0.0019144 |
| 208 | rs6748323 | T | C | T | C | -0.010359 | -0.013763 | NA | 0.537613 | 2 | 71101847 | 0.0170207 | 32072 | 0.418741 | 71101847 | 7.41E-09 | 2 | 389404 | 0.0017919 |
| 209 | rs67909753 | A | G | A | G | -0.01325 | -0.02239 | NA | 0.255685 | 5 | 173306058 | 0.0186649 | 32072 | 0.230311 | 173306058 | 1.58E-11 | 5 | 389404 | 0.0019657 |
| 210 | rs6803778 | T | G | T | G | -0.011172 | 0.0271177 | NA | 0.422898 | 3 | 132233917 | 0.0177469 | 32072 | 0.126506 | 132233917 | 2.27E-09 | 3 | 389404 | 0.0018692 |
| 211 | rs6857 | T | C | T | C | -0.015156 | -0.030139 | NA | 0.146206 | 19 | 45392254 | 0.0238129 | 32072 | 0.205637 | 45392254 | 1.30E-10 | 19 | 389404 | 0.0023579 |
| 212 | rs686364 | G | A | G | A | 0.0192663 | -0.009625 | NA | 0.282008 | 21 | 31587793 | 0.0193135 | 32072 | 0.618237 | 31587793 | 3.33E-20 | 21 | 389404 | 0.0020924 |
| 213 | rs6965823 | A | C | A | C | -0.01404 | -0.014558 | NA | 0.330434 | 7 | 4703360 | 0.019256 | 32072 | 0.449628 | 4703360 | 1.42E-13 | 7 | 389404 | 0.0018988 |
| 214 | rs6999484 | A | G | A | G | 0.0266321 | 0.0154808 | NA | 0.375438 | 8 | 23728271 | 0.0172551 | 32072 | 0.369626 | 23728271 | 2.83E-49 | 8 | 389404 | 0.0018049 |
| 215 | rs7126110 | G | C | G | C | 0.0469357 | -0.021352 | NA | 0.136938 | 11 | 64520255 | 0.0251556 | 32072 | 0.395994 | 64520255 | 6.26E-71 | 11 | 389404 | 0.0026358 |
| 216 | rs71359461 | C | G | C | G | 0.0125317 | 0.0156507 | NA | 0.483035 | 18 | 77156103 | 0.0182287 | 32072 | 0.390576 | 77156103 | 3.46E-12 | 18 | 389404 | 0.0018012 |
| 217 | rs7154553 | G | A | G | A | 0.0170087 | -0.036309 | NA | 0.183828 | 14 | 102686183 | 0.020869 | 32072 | 0.0818842 | 102686183 | 2.36E-14 | 14 | 389404 | 0.0022294 |
| 218 | rs7172166 | A | G | A | G | 0.0116031 | 0.0004142 | NA | 0.310115 | 15 | 81156959 | 0.0192175 | 32072 | 0.982803 | 81156959 | 1.30E-08 | 15 | 389404 | 0.0020405 |
| 219 | rs7179427 | A | G | A | G | 0.0167123 | -0.032364 | NA | 0.687913 | 15 | 72516821 | 0.0195376 | 32072 | 0.0976225 | 72516821 | 5.81E-17 | 15 | 389404 | 0.0019969 |
| 220 | rs7180968 | C | G | C | G | -0.010755 | -0.014238 | NA | 0.40528 | 15 | 39288527 | 0.0170959 | 32072 | 0.404951 | 39288527 | 2.14E-09 | 15 | 389404 | 0.0017966 |
| 221 | rs723585 | G | A | G | A | -0.010667 | 0.0318263 | NA | 0.44103 | 4 | 55503194 | 0.0172134 | 32072 | 0.0644689 | 55503194 | 7.12E-09 | 4 | 389404 | 0.0018429 |
| 222 | rs72683923 | C | T | C | T | -0.055078 | -0.171805 | NA | 0.0133025 | 14 | 50735947 | 0.0808245 | 32072 | 0.0335328 | 50735947 | 4.32E-18 | 14 | 389404 | 0.0063526 |
| 223 | rs72773715 | T | G | T | G | -0.024855 | -0.027918 | NA | 0.0302721 | 9 | 138970700 | 0.048333 | 32072 | 0.563518 | 138970700 | 2.52E-08 | 9 | 389404 | 0.0044608 |
| 224 | rs72777762 | G | C | G | C | -0.016101 | 0.0245057 | NA | 0.102004 | 10 | 12086214 | 0.0267676 | 32072 | 0.359929 | 12086214 | 1.06E-09 | 10 | 389404 | 0.0026395 |
| 225 | rs72799820 | T | C | T | C | -0.015862 | 0.0098002 | NA | 0.202504 | 16 | 71647450 | 0.0215821 | 32072 | 0.649765 | 71647450 | 1.96E-10 | 16 | 389404 | 0.0024922 |
| 226 | rs72818964 | A | G | A | G | 0.0160653 | 0.0027786 | NA | 0.140415 | 2 | 101578022 | 0.0231824 | 32072 | 0.904595 | 101578022 | 5.47E-12 | 2 | 389404 | 0.0023307 |
| 227 | rs73116829 | A | G | A | G | -0.021682 | 0.0126571 | NA | 0.0760352 | 7 | 50739738 | 0.0321231 | 32072 | 0.693566 | 50739738 | 3.49E-12 | 7 | 389404 | 0.0031168 |
| 228 | rs73153655 | G | A | G | A | 0.0131345 | -0.017313 | NA | 0.194444 | 12 | 84133370 | 0.0209348 | 32072 | 0.408238 | 84133370 | 5.86E-09 | 12 | 389404 | 0.0022565 |
| 229 | rs73169739 | C | T | C | T | 0.0221276 | -0.030312 | NA | 0.081929 | 3 | 170750018 | 0.0290206 | 32072 | 0.296262 | 170750018 | 3.10E-11 | 3 | 389404 | 0.0033317 |
| 230 | rs73225891 | G | C | G | C | -0.139732 | 0.0109192 | NA | 0.0215456 | 4 | 9922170 | 0.0750651 | 32072 | 0.884346 | 9922170 | 1.58E-134 | 4 | 389404 | 0.0056608 |
| 231 | rs738408 | T | C | T | C | -0.020636 | -0.006322 | NA | 0.252562 | 22 | 44324730 | 0.020252 | 32072 | 0.754918 | 44324730 | 9.53E-22 | 22 | 389404 | 0.0021536 |
| 232 | rs738794 | A | G | A | G | -0.018833 | -0.052979 | NA | 0.869548 | 22 | 24134758 | 0.0291432 | 32072 | 0.0690796 | 24134758 | 9.76E-09 | 22 | 389404 | 0.003284 |
| 233 | rs7402939 | C | T | C | T | 0.0115145 | -0.013581 | NA | 0.597072 | 15 | 99183876 | 0.0176338 | 32072 | 0.441203 | 99183876 | 5.20E-10 | 15 | 389404 | 0.0018533 |
| 234 | rs74606487 | G | A | G | A | -0.018953 | -0.018128 | NA | 0.119077 | 16 | 89795305 | 0.0269305 | 32072 | 0.50085 | 89795305 | 7.56E-14 | 16 | 389404 | 0.0025345 |
| 235 | rs74647166 | T | C | T | C | -0.026634 | -0.076561 | NA | 0.0295839 | 4 | 87477466 | 0.0511121 | 32072 | 0.13416 | 87477466 | 4.69E-08 | 4 | 389404 | 0.0048757 |
| 236 | rs7502296 | T | C | T | C | 0.0102818 | -0.008764 | NA | 0.327246 | 17 | 1614225 | 0.0192516 | 32072 | 0.648939 | 1614225 | 4.97E-08 | 17 | 389404 | 0.0018858 |
| 237 | rs7531171 | T | C | T | C | -0.010809 | 0.0180911 | NA | 0.68436 | 1 | 200264441 | 0.0180693 | 32072 | 0.316728 | 200264441 | 1.33E-08 | 1 | 389404 | 0.0019025 |
| 238 | rs7549723 | T | C | T | C | 0.0168716 | 0.0160039 | NA | 0.642125 | 1 | 150541812 | 0.0177057 | 32072 | 0.366056 | 150541812 | 1.41E-19 | 1 | 389404 | 0.0018639 |
| 239 | rs75588192 | A | G | A | G | 0.0224859 | -0.018236 | NA | 0.140065 | 12 | 133048600 | 0.0253548 | 32072 | 0.471992 | 133048600 | 8.83E-18 | 12 | 389404 | 0.0026182 |
| 240 | rs7563362 | G | A | G | A | 0.0161389 | 0.0345209 | NA | 0.844818 | 2 | 620297 | 0.0227713 | 32072 | 0.129524 | 620297 | 3.06E-10 | 2 | 389404 | 0.0025635 |
| 241 | rs7595982 | G | T | G | T | 0.0106409 | -0.022835 | NA | 0.455186 | 2 | 161339964 | 0.0177018 | 32072 | 0.197059 | 161332385 | 6.21E-09 | 2 | 389404 | 0.0018312 |
| 242 | rs760077 | T | A | T | A | -0.035153 | -0.001063 | NA | 0.624556 | 1 | 155178782 | 0.0176403 | 32072 | 0.951963 | 155178782 | 5.25E-83 | 1 | 389404 | 0.0018213 |
| 243 | rs7616014 | C | G | C | G | 0.0142854 | 0.0156985 | NA | 0.724909 | 3 | 114427057 | 0.0202888 | 32072 | 0.439076 | 114427057 | 4.33E-11 | 3 | 389404 | 0.002167 |
| 244 | rs76358556 | G | A | G | A | -0.018601 | -0.000438 | NA | 0.184184 | 3 | 126014827 | 0.0220599 | 32072 | 0.984165 | 126014827 | 3.31E-17 | 3 | 389404 | 0.0022053 |
| 245 | rs7650788 | C | T | C | T | -0.011029 | 0.0210397 | NA | 0.247008 | 3 | 64659758 | 0.0192305 | 32072 | 0.273918 | 64659758 | 1.50E-08 | 3 | 389404 | 0.0019479 |
| 246 | rs76612314 | A | G | A | G | 0.132329 | 0.0244556 | NA | 0.0690795 | 4 | 10209522 | 0.0347939 | 32072 | 0.482136 | 10209522 | 1.00E-200 | 4 | 389404 | 0.003453 |
| 247 | rs76850735 | C | T | C | T | 0.111747 | -0.209069 | NA | 0.0245478 | 4 | 10194377 | 0.051414 | 32072 | 4.77E-05 | 10194377 | 1.80E-107 | 4 | 389404 | 0.0050745 |
| 248 | rs76895963 | G | T | G | T | -0.057126 | 0.195316 | NA | 0.0159991 | 12 | 4384844 | 0.0667339 | 32072 | 0.0034248 | 4384844 | 1.08E-16 | 12 | 389404 | 0.0068863 |
| 249 | rs77542162 | G | A | G | A | -0.047346 | 0.110878 | NA | 0.0135945 | 17 | 67081278 | 0.0793593 | 32072 | 0.162363 | 67081278 | 1.36E-15 | 17 | 389404 | 0.0059266 |
| 250 | rs7773175 | G | C | G | C | -0.015753 | 0.0437066 | NA | 0.307598 | 6 | 31240959 | 0.018388 | 32072 | 0.0174582 | 31240959 | 7.52E-16 | 6 | 389404 | 0.0019541 |
| 251 | rs7779637 | G | A | G | A | -0.013732 | 0.0205421 | NA | 0.599434 | 7 | 128742164 | 0.0170489 | 32072 | 0.228246 | 128742164 | 1.81E-14 | 7 | 389404 | 0.001792 |
| 252 | rs78565962 | T | A | T | A | 0.0198041 | -0.050553 | NA | 0.0540041 | 13 | 50211276 | 0.0355667 | 32072 | 0.155209 | 50211276 | 3.06E-08 | 13 | 389404 | 0.0035762 |
| 253 | rs7893954 | G | A | G | A | -0.013226 | 0.0341032 | NA | 0.362221 | 10 | 104318966 | 0.0186075 | 32072 | 0.0668359 | 104318966 | 1.72E-11 | 10 | 389404 | 0.0019658 |
| 254 | rs7918123 | G | A | G | A | -0.009968 | -0.02907 | NA | 0.589544 | 10 | 52650502 | 0.0170987 | 32072 | 0.0891128 | 52650502 | 3.45E-08 | 10 | 389404 | 0.0018068 |
| 255 | rs7993948 | C | A | C | A | -0.017233 | 0.0224425 | NA | 0.851858 | 13 | 27629806 | 0.02526 | 32072 | 0.374293 | 27629806 | 7.68E-10 | 13 | 389404 | 0.0028015 |
| 256 | rs8019687 | A | T | A | T | -0.011209 | -0.010081 | NA | 0.706611 | 14 | 65956897 | 0.0184492 | 32072 | 0.584795 | 65951901 | 2.32E-09 | 14 | 389404 | 0.0018763 |
| 257 | rs8048364 | G | T | G | T | -0.013421 | 0.0099614 | NA | 0.180712 | 16 | 68253324 | 0.0218506 | 32072 | 0.648472 | 68253324 | 2.25E-08 | 16 | 389404 | 0.0024003 |
| 258 | rs8055680 | G | A | G | A | 0.0278727 | -0.021919 | NA | 0.0769077 | 16 | 24928922 | 0.0313818 | 32072 | 0.484884 | 24928922 | 8.04E-15 | 16 | 389404 | 0.0035886 |
| 259 | rs807624 | T | G | T | G | -0.019124 | 0.0223474 | NA | 0.413239 | 2 | 15782471 | 0.0178544 | 32072 | 0.210701 | 15782471 | 7.26E-25 | 2 | 389404 | 0.0018572 |
| 260 | rs8085083 | G | A | G | A | 0.0100972 | 0.0254427 | NA | 0.562271 | 18 | 40677365 | 0.0172005 | 32072 | 0.13909 | 40677365 | 2.31E-08 | 18 | 389404 | 0.0018074 |
| 261 | rs8103761 | A | C | A | C | -0.014555 | 0.0383563 | NA | 0.182774 | 19 | 1957517 | 0.0229098 | 32072 | 0.0940849 | 1957517 | 7.41E-10 | 19 | 389404 | 0.0023639 |
| 262 | rs833805 | G | A | G | A | 0.0233783 | 0.0289641 | NA | 0.887139 | 6 | 44030011 | 0.0269655 | 32072 | 0.28277 | 44030011 | 1.10E-15 | 6 | 389404 | 0.0029166 |
| 263 | rs856560 | C | T | C | T | 0.0152686 | -0.026355 | NA | 0.663647 | 7 | 46726963 | 0.0175744 | 32072 | 0.13371 | 46726963 | 9.69E-17 | 7 | 389404 | 0.0018377 |
| 264 | rs8614 | A | C | A | C | -0.013109 | 0.0057018 | NA | 0.227505 | 17 | 27588806 | 0.0224003 | 32072 | 0.799076 | 27588806 | 1.28E-08 | 17 | 389404 | 0.0023044 |
| 265 | rs879552 | A | G | A | G | -0.012468 | -0.036554 | NA | 0.364348 | 14 | 104010198 | 0.017695 | 32072 | 0.0388499 | 104010198 | 1.55E-11 | 14 | 389404 | 0.001849 |
| 266 | rs921385 | T | G | T | G | 0.0115261 | 0.039097 | NA | 0.261473 | 18 | 42784049 | 0.0186325 | 32072 | 0.0358765 | 42784049 | 2.50E-09 | 18 | 389404 | 0.0019335 |
| 267 | rs9260480 | C | T | C | T | -0.036353 | -0.000765 | NA | 0.050736 | 6 | 29920163 | 0.0395535 | 32072 | 0.984578 | 29920163 | 7.32E-15 | 6 | 389404 | 0.0046733 |
| 268 | rs9297949 | C | A | C | A | 0.019473 | -0.048334 | NA | 0.542039 | 8 | 95969445 | 0.017033 | 32072 | 0.0045442 | 95969445 | 6.91E-28 | 8 | 389404 | 0.0017789 |
| 269 | rs9300351 | A | G | A | G | -0.012196 | 0.0045344 | NA | 0.301126 | 13 | 97047639 | 0.0187515 | 32072 | 0.808924 | 97047639 | 6.80E-11 | 13 | 389404 | 0.0018691 |
| 270 | rs9480889 | G | C | G | C | 0.0133013 | -0.001092 | NA | 0.789173 | 6 | 109189021 | 0.0207292 | 32072 | 0.957977 | 109189021 | 7.24E-10 | 6 | 389404 | 0.0021591 |
| 271 | rs9534949 | G | C | G | C | -0.014976 | 0.0411691 | NA | 0.720995 | 13 | 48654455 | 0.0192898 | 32072 | 0.0328231 | 48654455 | 6.19E-14 | 13 | 389404 | 0.0019958 |
| 272 | rs963837 | C | T | C | T | -0.025142 | -0.002291 | NA | 0.448544 | 11 | 30749090 | 0.0169944 | 32072 | 0.892778 | 30749090 | 8.06E-45 | 11 | 389404 | 0.0017898 |
| 273 | rs968350 | A | G | A | G | -0.013873 | -0.046827 | NA | 0.696563 | 14 | 38070974 | 0.0181639 | 32072 | 0.0099362 | 38070974 | 4.53E-13 | 14 | 389404 | 0.0019166 |
| 274 | rs9704692 | T | C | T | C | 0.0147717 | 0.0371764 | NA | 0.40428 | 11 | 10368614 | 0.0169076 | 32072 | 0.0278927 | 10366029 | 3.79E-16 | 11 | 389404 | 0.0018135 |
| 275 | rs985062 | A | G | A | G | -0.011096 | -0.015482 | NA | 0.400177 | 7 | 24616474 | 0.0179397 | 32072 | 0.38814 | 24616474 | 4.30E-09 | 7 | 389404 | 0.0018895 |
| 276 | rs986267 | T | A | T | A | 0.0123468 | 0.0072444 | NA | 0.780155 | 6 | 131128242 | 0.0198143 | 32072 | 0.714653 | 131128242 | 2.97E-09 | 6 | 389404 | 0.002081 |
| 277 | rs9889162 | A | T | A | T | -0.011247 | -0.008905 | NA | 0.223321 | 16 | 56555788 | 0.019689 | 32072 | 0.651058 | 56555788 | 1.88E-08 | 16 | 389404 | 0.0020002 |
| 278 | rs9932625 | A | G | A | G | 0.0186663 | 0.0023347 | NA | 0.253973 | 16 | 51735746 | 0.0199883 | 32072 | 0.907014 | 51735746 | 1.14E-18 | 16 | 389404 | 0.0021162 |
| 279 | rs9996125 | A | G | A | G | -0.019963 | 0.0212499 | NA | 0.0811897 | 4 | 89285229 | 0.0330565 | 32072 | 0.520331 | 89285229 | 2.19E-09 | 4 | 389404 | 0.0033368 |
| 280 | rs10064782 | G | A | G | A | 0.015345 | -0.035284 | 0.25305 | 0.275721 | 5 | 67715161 | 0.019462 | 32072 | 0.0698377 | 67715161 | 6.28E-11 | 5 | 343836 | 0.0023473 |
| 281 | rs10160397 | C | T | C | T | 0.011442 | 0.0217333 | 0.39265 | 0.466765 | 11 | 61749766 | 0.0174371 | 32072 | 0.212623 | 61749766 | 3.90E-08 | 11 | 343836 | 0.002082 |
| 282 | rs10164318 | G | T | G | T | -0.019685 | 0.0351686 | 0.67313 | 0.642142 | 19 | 33459298 | 0.018104 | 32072 | 0.0520655 | 33459298 | 1.09E-19 | 19 | 343836 | 0.0021678 |
| 283 | rs10186643 | T | G | T | G | 0.019781 | 0.0160121 | 0.31406 | 0.368921 | 2 | 148803587 | 0.0180488 | 32072 | 0.374994 | 148803587 | 2.12E-19 | 2 | 343836 | 0.002196 |
| 284 | rs10193587 | C | T | C | T | -0.013484 | 0.0240011 | 0.23066 | 0.37357 | 2 | 136925439 | 0.0190164 | 32072 | 0.206905 | 136925439 | 3.09E-08 | 2 | 343836 | 0.0024355 |
| 285 | rs10196697 | A | G | A | G | -0.011756 | -0.033585 | 0.39092 | 0.471702 | 2 | 85700826 | 0.0171662 | 32072 | 0.0504139 | 85700826 | 1.73E-08 | 2 | 343836 | 0.0020855 |
| 286 | rs10210970 | T | C | T | C | 0.017682 | -0.03243 | 0.12894 | 0.261399 | 2 | 28646847 | 0.022611 | 32072 | 0.151498 | 28646847 | 5.45E-09 | 2 | 343836 | 0.0030314 |
| 287 | rs10224210 | C | T | C | T | 0.026693 | -0.01137 | 0.28247 | 0.249653 | 7 | 151413194 | 0.0186988 | 32072 | 0.54314 | 151413194 | 3.27E-32 | 7 | 343836 | 0.002259 |
| 288 | rs10405423 | A | C | A | C | 0.023016 | 0.015217 | 0.65781 | 0.692595 | 19 | 7211311 | 0.018016 | 32072 | 0.398313 | 7211311 | 2.10E-26 | 19 | 343836 | 0.0021644 |
| 289 | rs1047891 | A | C | A | C | -0.027571 | -0.011325 | 0.31573 | 0.295502 | 2 | 211540507 | 0.018372 | 32072 | 0.537617 | 211540507 | 1.82E-36 | 2 | 343836 | 0.0021858 |
| 290 | rs10754894 | A | G | A | G | -0.021522 | 0.0024441 | 0.31406 | 0.331738 | 1 | 15835234 | 0.0185738 | 32072 | 0.895308 | 15835234 | 7.44E-23 | 1 | 343836 | 0.0021866 |
| 291 | rs10782230 | A | G | A | G | 0.01481 | -0.023343 | 0.48093 | 0.486602 | 6 | 126228512 | 0.0169889 | 32072 | 0.169431 | 126228512 | 3.35E-13 | 6 | 343836 | 0.0020344 |
| 292 | rs10797999 | T | C | T | C | -0.013044 | -0.006819 | 0.41098 | 0.410834 | 1 | 185137628 | 0.017343 | 32072 | 0.694202 | 185137628 | 2.97E-10 | 1 | 343836 | 0.0020703 |
| 293 | rs10846156 | G | T | G | T | -0.016485 | -0.012746 | 0.20201 | 0.213829 | 12 | 15321921 | 0.0212529 | 32072 | 0.548698 | 15321921 | 8.18E-11 | 12 | 343836 | 0.0025371 |
| 294 | rs10886117 | A | G | A | G | 0.020193 | 0.0232689 | 0.16706 | 0.173923 | 10 | 119480578 | 0.0244198 | 32072 | 0.340654 | 119480578 | 1.39E-13 | 10 | 343836 | 0.0027298 |
| 295 | rs10935686 | C | T | C | T | -0.012627 | -0.001547 | 0.56061 | 0.590381 | 3 | 94048174 | 0.0171236 | 32072 | 0.928034 | 94048174 | 6.75E-10 | 3 | 343836 | 0.0020458 |
| 296 | rs10958567 | G | C | G | C | -0.015961 | -0.040437 | 0.24463 | 0.239855 | 8 | 38108133 | 0.020507 | 32072 | 0.0486262 | 38108133 | 1.39E-11 | 8 | 343836 | 0.0023613 |
| 297 | rs11072567 | G | A | G | A | 0.030702 | 0.0526676 | 0.51215 | 0.521499 | 15 | 76298744 | 0.0172963 | 32072 | 0.0023266 | 76298744 | 1.52E-51 | 15 | 343836 | 0.0020323 |
| 298 | rs11108219 | C | T | C | T | 0.017086 | -0.002089 | 0.22117 | 0.173242 | 12 | 96121902 | 0.0215876 | 32072 | 0.92291 | 96121902 | 3.11E-12 | 12 | 343836 | 0.0024503 |
| 299 | rs11128603 | G | A | G | A | -0.021478 | -0.01831 | 0.12134 | 0.106055 | 3 | 12385828 | 0.0267428 | 32072 | 0.493558 | 12385828 | 4.84E-12 | 3 | 343836 | 0.0031081 |
| 300 | rs111295548 | G | A | G | A | -0.031415 | 0.0433774 | 0.034229 | 0.0311619 | 1 | 186806345 | 0.0487038 | 32072 | 0.373124 | 186806345 | 2.02E-08 | 1 | 343836 | 0.0055998 |
| 301 | rs111346856 | A | G | A | G | 0.013965 | 0.0175199 | 0.27703 | 0.214677 | 10 | 65193018 | 0.0199869 | 32072 | 0.38072 | 65193018 | 7.94E-10 | 10 | 343836 | 0.0022721 |
| 302 | rs11163481 | T | G | T | G | 0.013343 | -0.009739 | 0.67037 | 0.672721 | 1 | 82937433 | 0.0185674 | 32072 | 0.599907 | 82937433 | 7.43E-10 | 1 | 343836 | 0.0021671 |
| 303 | rs11202328 | T | C | T | C | -0.025529 | -0.032897 | 0.13132 | 0.18401 | 10 | 88845190 | 0.0274783 | 32072 | 0.231233 | 88845190 | 2.39E-17 | 10 | 343836 | 0.0030128 |
| 304 | rs11218783 | A | G | A | G | 0.011414 | 0.0061038 | 0.43061 | 0.39564 | 11 | 122603367 | 0.0171244 | 32072 | 0.721511 | 122603367 | 2.94E-08 | 11 | 343836 | 0.0020585 |
| 305 | rs114165349 | C | G | C | G | 0.080523 | 0.0709248 | 0.023533 | 0.0181488 | 1 | 27021913 | 0.0607334 | 32072 | 0.242885 | 27021913 | 8.13E-33 | 1 | 343836 | 0.0067482 |
| 306 | rs11564722 | T | C | T | C | -0.026087 | -0.006127 | 0.23803 | 0.320332 | 11 | 2178330 | 0.0190116 | 32072 | 0.747228 | 2178330 | 3.74E-27 | 11 | 343836 | 0.002417 |
| 307 | rs11600628 | T | C | T | C | -0.031211 | 0.0068115 | 0.17498 | 0.141078 | 11 | 63976027 | 0.0245833 | 32072 | 0.78172 | 63976027 | 1.49E-31 | 11 | 343836 | 0.0026704 |
| 308 | rs116402366 | A | G | A | G | 0.055468 | -0.003426 | 0.025027 | 0.0200741 | 5 | 176790807 | 0.0595751 | 32072 | 0.954142 | 176790807 | 8.22E-17 | 5 | 343836 | 0.00666 |
| 309 | rs11671389 | T | A | T | A | 0.016764 | -0.042107 | 0.25404 | 0.246367 | 19 | 18210922 | 0.0197376 | 32072 | 0.0328965 | 18210922 | 8.22E-13 | 19 | 343836 | 0.0023421 |
| 310 | rs11693363 | C | A | C | A | -0.030537 | 0.004127 | 0.13087 | 0.104969 | 2 | 69821008 | 0.027326 | 32072 | 0.879954 | 69821008 | 3.89E-24 | 2 | 343836 | 0.003013 |
| 311 | rs1171614 | C | T | C | T | 0.05629 | -0.032025 | 0.76883 | 0.804543 | 10 | 61469538 | 0.0202861 | 32072 | 0.114408 | 61469538 | 2.14E-120 | 10 | 343836 | 0.0024117 |
| 312 | rs11748431 | A | G | A | G | -0.012762 | -0.008448 | 0.2619 | 0.223425 | 5 | 180068741 | 0.0193563 | 32072 | 0.662518 | 180068741 | 3.95E-08 | 5 | 343836 | 0.0023233 |
| 313 | rs11765552 | A | T | A | T | 0.015398 | 0.0226254 | 0.53662 | 0.492029 | 7 | 97822115 | 0.0170846 | 32072 | 0.185399 | 97822115 | 3.71E-14 | 7 | 343836 | 0.0020338 |
| 314 | rs11835818 | C | T | C | T | -0.018637 | 0.0042137 | 0.47895 | 0.497279 | 12 | 122494809 | 0.0171528 | 32072 | 0.805949 | 122494809 | 5.83E-20 | 12 | 343836 | 0.0020373 |
| 315 | rs11854957 | T | C | T | C | -0.014368 | 0.0300318 | 0.21878 | 0.1742 | 15 | 74467796 | 0.0217993 | 32072 | 0.168312 | 74467796 | 4.86E-09 | 15 | 343836 | 0.0024552 |
| 316 | rs1186705 | C | A | C | A | -0.013628 | 0.0055192 | 0.3475 | 0.392791 | 2 | 61710573 | 0.0176067 | 32072 | 0.753921 | 61673278 | 2.00E-10 | 2 | 343836 | 0.0021423 |
| 317 | rs12133907 | A | C | A | C | 0.01917 | -0.029724 | 0.6023 | 0.614003 | 1 | 93850283 | 0.0176135 | 32072 | 0.0914892 | 93850283 | 2.36E-20 | 1 | 343836 | 0.0020735 |
| 318 | rs12144369 | T | C | T | C | -0.012046 | 0.0054194 | 0.35829 | 0.373859 | 1 | 227254045 | 0.0175985 | 32072 | 0.758125 | 227254045 | 2.58E-08 | 1 | 343836 | 0.0021635 |
| 319 | rs1229984 | C | T | C | T | -0.060041 | 0.0019077 | 0.977726 | 0.82244 | 4 | 100239319 | 0.0323506 | 32072 | 0.952976 | 100239319 | 2.84E-18 | 4 | 343836 | 0.006887 |
| 320 | rs12316443 | A | C | A | C | -0.015118 | -0.003498 | 0.30511 | 0.266914 | 12 | 78810733 | 0.0187547 | 32072 | 0.85204 | 78810733 | 1.03E-11 | 12 | 343836 | 0.0022226 |
| 321 | rs12363578 | T | C | T | C | -0.055638 | -0.008792 | 0.4301 | 0.362794 | 11 | 64364866 | 0.0178651 | 32072 | 0.622621 | 64364866 | 6.85E-159 | 11 | 343836 | 0.0020705 |
| 322 | rs12371604 | C | T | C | T | -0.014317 | -0.008976 | 0.21843 | 0.309271 | 12 | 21391336 | 0.0198893 | 32072 | 0.651775 | 21391336 | 5.71E-09 | 12 | 343836 | 0.0024578 |
| 323 | rs12443147 | T | C | T | C | -0.014781 | 0.0379396 | 0.2135 | 0.241198 | 15 | 50983543 | 0.0210802 | 32072 | 0.0718952 | 50983543 | 3.06E-09 | 15 | 343836 | 0.0024933 |
| 324 | rs12554192 | G | A | G | A | 0.014457 | -0.044676 | 0.26969 | 0.257972 | 9 | 33084732 | 0.0188167 | 32072 | 0.0175837 | 33084732 | 2.69E-10 | 9 | 343836 | 0.0022891 |
| 325 | rs12576996 | G | T | G | T | 0.037505 | -0.006842 | 0.24609 | 0.253333 | 11 | 65580638 | 0.0189521 | 32072 | 0.718098 | 65580638 | 2.74E-56 | 11 | 343836 | 0.0023721 |
| 326 | rs12602520 | C | A | C | A | 0.020274 | 0.0384366 | 0.086525 | 0.106479 | 17 | 26955330 | 0.0290221 | 32072 | 0.185374 | 26955330 | 1.99E-08 | 17 | 343836 | 0.0036121 |
| 327 | rs1260326 | C | T | C | T | -0.049025 | 0.0336799 | 0.60689 | 0.565036 | 2 | 27730940 | 0.0174566 | 32072 | 0.0536883 | 27730940 | 5.45E-123 | 2 | 343836 | 0.0020778 |
| 328 | rs12697963 | T | C | T | C | 0.014496 | -0.00239 | 0.72073 | 0.679121 | 7 | 156249815 | 0.0189936 | 32072 | 0.899873 | 156249815 | 1.91E-10 | 7 | 343836 | 0.002276 |
| 329 | rs12708477 | C | A | C | A | -0.014043 | 0.0147469 | 0.77225 | 0.780812 | 15 | 63605954 | 0.0199482 | 32072 | 0.459749 | 63605954 | 7.06E-09 | 15 | 343836 | 0.0024256 |
| 330 | rs1285875 | C | G | C | G | -0.040214 | 0.0153489 | 0.74254 | 0.643922 | 6 | 7115927 | 0.0194575 | 32072 | 0.430205 | 7115927 | 7.89E-67 | 6 | 343836 | 0.002328 |
| 331 | rs12882763 | T | C | T | C | -0.011882 | -0.00918 | 0.66188 | 0.696382 | 14 | 66110628 | 0.0180776 | 32072 | 0.611582 | 66078025 | 3.33E-08 | 14 | 343836 | 0.0021513 |
| 332 | rs1294448 | G | C | G | C | 0.012674 | -0.035085 | 0.50489 | 0.528735 | 6 | 6727363 | 0.017525 | 32072 | 0.0452877 | 6727363 | 5.47E-10 | 6 | 343836 | 0.0020424 |
| 333 | rs13107325 | T | C | T | C | -0.031043 | 0.025279 | 0.074844 | 0.0674602 | 4 | 103188709 | 0.0317715 | 32072 | 0.426237 | 103188709 | 1.00E-15 | 4 | 343836 | 0.0038672 |
| 334 | rs13130003 | G | A | G | A | -0.038018 | -0.028775 | 0.72499 | 0.725697 | 4 | 10113047 | 0.0197259 | 32072 | 0.144637 | 10113047 | 3.01E-62 | 4 | 343836 | 0.0022829 |
| 335 | rs13153909 | A | T | A | T | 0.014124 | -0.001555 | 0.67279 | 0.651661 | 5 | 55446970 | 0.0184905 | 32072 | 0.932977 | 55446970 | 1.05E-10 | 5 | 343836 | 0.0021863 |
| 336 | rs1317983 | C | T | C | T | 0.031692 | 0.0164725 | 0.69388 | 0.718575 | 6 | 43806335 | 0.0185118 | 32072 | 0.373554 | 43806335 | 5.57E-47 | 6 | 343836 | 0.0022013 |
| 337 | rs13198778 | T | C | T | C | -0.011568 | 0.010233 | 0.36381 | 0.374053 | 6 | 42751778 | 0.0180214 | 32072 | 0.570152 | 42751778 | 4.39E-08 | 6 | 343836 | 0.0021131 |
| 338 | rs13240994 | C | T | C | T | -0.034945 | -0.001536 | 0.19956 | 0.173625 | 7 | 73016862 | 0.0217755 | 32072 | 0.943755 | 73016862 | 5.09E-43 | 7 | 343836 | 0.0025411 |
| 339 | rs13411042 | A | C | A | C | 0.01667 | -0.047793 | 0.48462 | 0.494956 | 2 | 183087067 | 0.0171026 | 32072 | 0.0051988 | 183087067 | 2.63E-16 | 2 | 343836 | 0.0020356 |
| 340 | rs138660816 | A | G | A | G | 0.054056 | 0.120396 | 0.01232 | 0.0076799 | 4 | 9701572 | 0.12145 | 32072 | 0.32153 | 9701572 | 2.88E-08 | 4 | 343836 | 0.0097423 |
| 341 | rs140254647 | C | T | C | T | -0.01576 | -0.034494 | 0.21545 | 0.203363 | 1 | 48875669 | 0.0202219 | 32072 | 0.0880501 | 48875669 | 2.09E-10 | 1 | 343836 | 0.00248 |
| 342 | rs141471965 | T | C | T | C | 0.17118 | -0.056416 | 0.1145 | 0.123946 | 4 | 89046202 | 0.0286986 | 32072 | 0.0493208 | 89046202 | 1.00E-200 | 4 | 343836 | 0.0031767 |
| 343 | rs1440411 | T | C | T | C | -0.015339 | 0.0311384 | 0.57316 | 0.615063 | 4 | 144158285 | 0.0172683 | 32072 | 0.0713543 | 144158285 | 1.06E-13 | 4 | 343836 | 0.0020636 |
| 344 | rs1472866 | A | G | A | G | -0.01165 | -0.006071 | 0.5016 | 0.583694 | 4 | 146849715 | 0.0174412 | 32072 | 0.727797 | 146849715 | 1.20E-08 | 4 | 343836 | 0.0020437 |
| 345 | rs148179165 | A | C | A | C | 0.016243 | 0.0146143 | 0.30611 | 0.330368 | 12 | 52252338 | 0.0184797 | 32072 | 0.429041 | 52249232 | 2.59E-13 | 12 | 343836 | 0.0022206 |
| 346 | rs148185902 | A | G | A | G | 0.063469 | -0.002778 | 0.013808 | 0.0099543 | 11 | 30718534 | 0.092278 | 32072 | 0.97598 | 30718534 | 5.43E-12 | 11 | 343836 | 0.0092061 |
| 347 | rs150147865 | T | A | T | A | -0.11629 | -0.054939 | 0.0088936 | 0.0116025 | 1 | 120326253 | 0.0902956 | 32072 | 0.542898 | 120326253 | 8.75E-24 | 1 | 343836 | 0.011565 |
| 348 | rs1541939 | G | T | G | T | 0.014222 | -0.000409 | 0.25549 | 0.357908 | 11 | 5578698 | 0.0189308 | 32072 | 0.982776 | 5578434 | 1.18E-09 | 11 | 343836 | 0.002338 |
| 349 | rs16890979 | T | C | T | C | -0.3122 | 0.0517716 | 0.22331 | 0.193041 | 4 | 9922167 | 0.0207406 | 32072 | 0.0125548 | 9922167 | 1.00E-200 | 4 | 343836 | 0.0023151 |
| 350 | rs16951334 | G | A | G | A | 0.02156 | 0.0325026 | 0.10671 | 0.103795 | 13 | 96407088 | 0.0270891 | 32072 | 0.230201 | 96407088 | 6.01E-11 | 13 | 343836 | 0.0032948 |
| 351 | rs17024258 | T | C | T | C | 0.035978 | 0.0003706 | 0.02586 | 0.0234817 | 1 | 110147321 | 0.051699 | 32072 | 0.994281 | 110147321 | 2.12E-08 | 1 | 343836 | 0.0064227 |
| 352 | rs17050272 | A | G | A | G | 0.025372 | 0.016833 | 0.40966 | 0.418572 | 2 | 121306440 | 0.0173439 | 32072 | 0.331776 | 121306440 | 1.22E-34 | 2 | 343836 | 0.0020666 |
| 353 | rs1705614 | T | C | T | C | 0.022165 | 0.0062987 | 0.72452 | 0.737292 | 3 | 141811257 | 0.0194739 | 32072 | 0.746361 | 141811257 | 2.12E-22 | 3 | 343836 | 0.0022765 |
| 354 | rs17477449 | C | G | C | G | -0.19945 | 0.075451 | 0.016335 | 0.0177912 | 4 | 10313388 | 0.0675522 | 32072 | 0.264025 | 10313388 | 8.15E-137 | 4 | 343836 | 0.0080074 |
| 355 | rs17624477 | C | T | C | T | 0.027214 | -0.019232 | 0.063891 | 0.0681414 | 16 | 24955183 | 0.0322679 | 32072 | 0.551169 | 24955183 | 5.73E-11 | 16 | 343836 | 0.0041542 |
| 356 | rs17632159 | C | G | C | G | -0.033908 | -0.013909 | 0.30419 | 0.292685 | 5 | 72431482 | 0.0187926 | 32072 | 0.459238 | 72431482 | 6.33E-53 | 5 | 343836 | 0.002214 |
| 357 | rs17817497 | C | T | C | T | 0.017787 | -0.004992 | 0.39284 | 0.373675 | 16 | 53815435 | 0.0172257 | 32072 | 0.771983 | 53815435 | 1.34E-17 | 16 | 343836 | 0.0020826 |
| 358 | rs1800961 | T | C | T | C | -0.050379 | -0.054688 | 0.031412 | 0.027395 | 20 | 43042364 | 0.0483095 | 32072 | 0.25762 | 43042364 | 5.23E-18 | 20 | 343836 | 0.005825 |
| 359 | rs181673 | C | A | C | A | 0.018486 | -0.017559 | 0.53613 | 0.605307 | 10 | 119388528 | 0.0172767 | 32072 | 0.309465 | 119388528 | 1.06E-19 | 10 | 343836 | 0.0020351 |
| 360 | rs1869581 | C | G | C | G | -0.011565 | 0.0125463 | 0.49078 | 0.423763 | 8 | 126558350 | 0.0182085 | 32072 | 0.490801 | 126558350 | 1.97E-08 | 8 | 343836 | 0.0020597 |
| 361 | rs187355703 | G | C | G | C | 0.057634 | -0.071465 | 0.025703 | 0.0166351 | 2 | 176993583 | 0.0626515 | 32072 | 0.254006 | 176993583 | 4.71E-19 | 2 | 343836 | 0.0064616 |
| 362 | rs193220 | T | C | T | C | -0.015791 | 0.0233211 | 0.31734 | 0.367066 | 17 | 74268619 | 0.0182073 | 32072 | 0.20024 | 74268619 | 5.20E-13 | 17 | 343836 | 0.0021871 |
| 363 | rs1949651 | T | C | T | C | 0.012775 | -0.007227 | 0.47115 | 0.535136 | 2 | 213117629 | 0.0171586 | 32072 | 0.673605 | 213117629 | 4.23E-10 | 2 | 343836 | 0.0020454 |
| 364 | rs195485 | T | C | T | C | -0.013006 | 0.0032601 | 0.38469 | 0.336703 | 6 | 116232098 | 0.0178099 | 32072 | 0.854759 | 116232098 | 5.35E-10 | 6 | 343836 | 0.0020949 |
| 365 | rs1965132 | A | C | A | C | 0.015524 | -0.01625 | 0.49442 | 0.50283 | 3 | 69147519 | 0.0170185 | 32072 | 0.339649 | 69147519 | 3.75E-14 | 3 | 343836 | 0.0020508 |
| 366 | rs1991371 | A | G | A | G | 0.025986 | 0.0037523 | 0.11941 | 0.106178 | 2 | 203418537 | 0.027019 | 32072 | 0.889547 | 203418537 | 1.12E-16 | 2 | 343836 | 0.003134 |
| 367 | rs2108093 | G | A | G | A | 0.015625 | 0.033505 | 0.79835 | 0.841561 | 22 | 30679387 | 0.0225595 | 32072 | 0.137494 | 30679387 | 7.02E-10 | 22 | 343836 | 0.0025341 |
| 368 | rs2195525 | T | C | T | C | -0.014963 | 0.0100672 | 0.52068 | 0.5748 | 11 | 119235404 | 0.0170474 | 32072 | 0.554826 | 119235404 | 3.21E-13 | 11 | 343836 | 0.0020537 |
| 369 | rs219787 | T | C | T | C | -0.022544 | -0.019104 | 0.2628 | 0.271697 | 21 | 37829508 | 0.0202701 | 32072 | 0.345954 | 37829508 | 1.66E-22 | 21 | 343836 | 0.0023095 |
| 370 | rs2229357 | A | G | A | G | -0.048044 | 0.0147946 | 0.24372 | 0.199755 | 12 | 57843711 | 0.0207929 | 32072 | 0.476762 | 57843711 | 8.53E-92 | 12 | 343836 | 0.0023637 |
| 371 | rs2240390 | C | T | C | T | -0.014384 | 0.0108669 | 0.3936 | 0.373383 | 7 | 101257075 | 0.017497 | 32072 | 0.534555 | 101257075 | 6.02E-12 | 7 | 343836 | 0.0020908 |
| 372 | rs2244552 | G | A | G | A | 0.032696 | -0.038748 | 0.42698 | 0.421673 | 3 | 53055522 | 0.0172154 | 32072 | 0.0243994 | 53055522 | 4.83E-57 | 3 | 343836 | 0.0020538 |
| 373 | rs2258043 | C | T | C | T | -0.013075 | 0.0378863 | 0.49006 | 0.525982 | 12 | 121451425 | 0.0175019 | 32072 | 0.0304103 | 121451425 | 1.31E-10 | 12 | 343836 | 0.0020345 |
| 374 | rs2269863 | T | C | T | C | 0.013639 | -0.007307 | 0.59176 | 0.493747 | 16 | 20857260 | 0.0172975 | 32072 | 0.672729 | 20857260 | 4.07E-11 | 16 | 343836 | 0.002066 |
| 375 | rs2345962 | G | A | G | A | 0.015173 | 0.0089736 | 0.49877 | 0.514367 | 1 | 163741788 | 0.0174544 | 32072 | 0.60717 | 163741788 | 9.88E-14 | 1 | 343836 | 0.0020386 |
| 376 | rs2394685 | G | A | G | A | -0.015068 | 0.0073195 | 0.51303 | 0.543646 | 6 | 29745439 | 0.0170286 | 32072 | 0.667315 | 29745439 | 1.30E-13 | 6 | 343836 | 0.0020343 |
| 377 | rs2436958 | C | T | C | T | -0.021263 | -0.039511 | 0.85661 | 0.866189 | 8 | 103641791 | 0.0257681 | 32072 | 0.125194 | 103641791 | 2.14E-13 | 8 | 343836 | 0.0028968 |
| 378 | rs2437817 | A | C | A | C | -0.015239 | 0.0120439 | 0.33016 | 0.353988 | 9 | 107690057 | 0.017587 | 32072 | 0.49346 | 107690057 | 2.54E-12 | 9 | 343836 | 0.0021765 |
| 379 | rs2439823 | G | A | G | A | 0.011672 | -0.003732 | 0.54673 | 0.500553 | 10 | 99778226 | 0.0172191 | 32072 | 0.828418 | 99778226 | 1.19E-08 | 10 | 343836 | 0.0020474 |
| 380 | rs2480714 | T | G | T | G | 0.013719 | -0.026316 | 0.69836 | 0.726118 | 1 | 2144982 | 0.0184775 | 32072 | 0.154388 | 2144982 | 9.82E-10 | 1 | 343836 | 0.0022443 |
| 381 | rs2540034 | T | C | T | C | 0.011723 | -0.010436 | 0.57106 | 0.524891 | 16 | 4022694 | 0.0174022 | 32072 | 0.548713 | 4022694 | 1.98E-08 | 16 | 343836 | 0.0020881 |
| 382 | rs255751 | T | C | T | C | 0.019229 | -0.008803 | 0.69907 | 0.703521 | 5 | 53319517 | 0.018161 | 32072 | 0.627869 | 53319517 | 5.05E-18 | 5 | 343836 | 0.0022224 |
| 383 | rs2636590 | A | G | A | G | 0.011581 | -0.001978 | 0.61313 | 0.615657 | 6 | 133935728 | 0.0174382 | 32072 | 0.909699 | 133935728 | 2.97E-08 | 6 | 343836 | 0.0020891 |
| 384 | rs2644128 | G | C | G | C | 0.014378 | 0.0355292 | 0.55138 | 0.474351 | 1 | 201793440 | 0.0171205 | 32072 | 0.0379638 | 201793440 | 2.05E-12 | 1 | 343836 | 0.0020448 |
| 385 | rs2688 | T | G | T | G | -0.011457 | 0.0040355 | 0.60656 | 0.614082 | 17 | 36046931 | 0.0175477 | 32072 | 0.818113 | 36046931 | 3.91E-08 | 17 | 343836 | 0.0020851 |
| 386 | rs2788144 | G | A | G | A | 0.039458 | -0.007032 | 0.03673 | 0.0287028 | 1 | 212069929 | 0.0484553 | 32072 | 0.884608 | 212069929 | 3.39E-13 | 1 | 343836 | 0.0054213 |
| 387 | rs2817188 | G | A | G | A | 0.066981 | -0.013033 | 0.56825 | 0.581394 | 6 | 25807603 | 0.016988 | 32072 | 0.442984 | 25807603 | 1.00E-200 | 6 | 343836 | 0.0020493 |
| 388 | rs2823139 | A | G | A | G | 0.015734 | 0.0181909 | 0.33841 | 0.314145 | 21 | 16576783 | 0.018143 | 32072 | 0.316033 | 16576783 | 3.05E-13 | 21 | 343836 | 0.0021577 |
| 389 | rs2834317 | A | G | A | G | 0.019118 | -0.006343 | 0.15374 | 0.134273 | 21 | 35356706 | 0.0244617 | 32072 | 0.795404 | 35356706 | 1.58E-11 | 21 | 343836 | 0.0028363 |
| 390 | rs28371924 | T | G | T | G | -0.012531 | -0.003772 | 0.498 | 0.528656 | 5 | 68746878 | 0.0176094 | 32072 | 0.830372 | 68746878 | 9.59E-10 | 5 | 343836 | 0.0020489 |
| 391 | rs28384289 | T | C | T | C | 0.028528 | 0.0172755 | 0.14729 | 0.150242 | 16 | 79925400 | 0.0245377 | 32072 | 0.481408 | 79925400 | 2.16E-23 | 16 | 343836 | 0.0028625 |
| 392 | rs28517717 | T | C | T | C | -0.022953 | -0.014295 | 0.27454 | 0.319076 | 9 | 33179451 | 0.0191619 | 32072 | 0.45567 | 33179451 | 1.66E-23 | 9 | 343836 | 0.0022971 |
| 393 | rs28848873 | G | C | G | C | 0.012675 | 0.0067178 | 0.27767 | 0.339272 | 3 | 156927413 | 0.0186647 | 32072 | 0.718906 | 156916920 | 4.56E-08 | 3 | 343836 | 0.0023181 |
| 394 | rs288749 | C | G | C | G | 0.012549 | 0.0351508 | 0.65831 | 0.706697 | 7 | 155620122 | 0.0180473 | 32072 | 0.0514494 | 155620122 | 4.99E-09 | 7 | 343836 | 0.0021459 |
| 395 | rs2941484 | T | C | T | C | 0.031849 | 0.039503 | 0.44541 | 0.43962 | 8 | 76478768 | 0.0170951 | 32072 | 0.0208449 | 76478768 | 4.05E-54 | 8 | 343836 | 0.0020557 |
| 396 | rs2943654 | T | C | T | C | 0.013195 | 0.0250138 | 0.64567 | 0.688891 | 2 | 227112754 | 0.0177728 | 32072 | 0.159302 | 227112754 | 5.20E-10 | 2 | 343836 | 0.0021237 |
| 397 | rs2973444 | C | T | C | T | -0.025163 | -0.02432 | 0.085758 | 0.0796827 | 5 | 90417593 | 0.0299214 | 32072 | 0.416345 | 90417593 | 4.88E-12 | 5 | 343836 | 0.003642 |
| 398 | rs3184504 | C | T | C | T | -0.02881 | 0.075341 | 0.51798 | 0.579867 | 12 | 111884608 | 0.0170966 | 32072 | 1.05E-05 | 111884608 | 1.40E-45 | 12 | 343836 | 0.0020329 |
| 399 | rs33938520 | T | C | T | C | -0.01345 | 0.0128334 | 0.25276 | 0.249743 | 19 | 4984588 | 0.0187112 | 32072 | 0.492797 | 4984588 | 9.79E-09 | 19 | 343836 | 0.0023455 |
| 400 | rs345748 | G | A | G | A | 0.015091 | 0.0184172 | 0.75257 | 0.701316 | 15 | 33343118 | 0.0195082 | 32072 | 0.345133 | 33343118 | 1.46E-10 | 15 | 343836 | 0.0023543 |
| 401 | rs34811474 | A | G | A | G | -0.013991 | -0.023968 | 0.23213 | 0.191584 | 4 | 25408838 | 0.0227649 | 32072 | 0.292402 | 25408838 | 6.04E-09 | 4 | 343836 | 0.0024057 |
| 402 | rs35016056 | G | T | G | T | 0.013501 | -0.016699 | 0.54977 | 0.442504 | 17 | 45440269 | 0.0170028 | 32072 | 0.326023 | 45425780 | 4.19E-11 | 17 | 343836 | 0.0020464 |
| 403 | rs35184622 | G | A | G | A | 0.013967 | 0.0223948 | 0.32728 | 0.316171 | 15 | 86073035 | 0.017787 | 32072 | 0.20801 | 86073035 | 5.20E-10 | 15 | 343836 | 0.0022481 |
| 404 | rs354532 | T | C | T | C | 0.012135 | -0.005003 | 0.53859 | 0.546625 | 6 | 111548813 | 0.0172226 | 32072 | 0.771438 | 111548813 | 3.01E-09 | 6 | 343836 | 0.0020459 |
| 405 | rs358226 | G | A | G | A | 0.017849 | 0.0302409 | 0.85401 | 0.828886 | 4 | 22825055 | 0.0237423 | 32072 | 0.202765 | 22825055 | 5.43E-10 | 4 | 343836 | 0.0028759 |
| 406 | rs36071802 | C | T | C | T | 0.025477 | 0.0153801 | 0.41557 | 0.374714 | 8 | 23719571 | 0.0172549 | 32072 | 0.372743 | 23715871 | 5.98E-34 | 8 | 343836 | 0.0020972 |
| 407 | rs3746574 | C | T | C | T | -0.014949 | -0.034235 | 0.51529 | 0.51492 | 20 | 43058018 | 0.0177035 | 32072 | 0.0531374 | 43058018 | 5.43E-13 | 20 | 343836 | 0.0020721 |
| 408 | rs3824359 | C | T | C | T | 0.019211 | -0.006688 | 0.14193 | 0.166095 | 9 | 139105229 | 0.0238475 | 32072 | 0.779123 | 139105229 | 4.15E-11 | 9 | 343836 | 0.0029112 |
| 409 | rs3925584 | C | T | C | T | -0.025448 | -0.000958 | 0.45463 | 0.443328 | 11 | 30760335 | 0.0169739 | 32072 | 0.955005 | 30760335 | 8.49E-36 | 11 | 343836 | 0.0020373 |
| 410 | rs40270 | C | A | C | A | 0.020959 | 0.0332007 | 0.77305 | 0.68077 | 5 | 55804552 | 0.0200966 | 32072 | 0.0985213 | 55804552 | 7.33E-17 | 5 | 343836 | 0.0025124 |
| 411 | rs41264869 | T | C | T | C | 0.016214 | 0.0412827 | 0.17848 | 0.199794 | 1 | 205030862 | 0.0228228 | 32072 | 0.0704774 | 205030862 | 1.02E-09 | 1 | 343836 | 0.0026552 |
| 412 | rs41290716 | A | C | A | C | 0.035631 | -0.009294 | 0.070006 | 0.0532376 | 3 | 49717795 | 0.0365326 | 32072 | 0.799188 | 49717795 | 3.43E-19 | 3 | 343836 | 0.0039791 |
| 413 | rs4140872 | T | C | T | C | -0.0282 | 0.0020222 | 0.75583 | 0.713065 | 2 | 170026596 | 0.0198487 | 32072 | 0.918851 | 170026596 | 1.04E-32 | 2 | 343836 | 0.0023674 |
| 414 | rs423144 | T | G | T | G | -0.036155 | 0.0244725 | 0.42619 | 0.476346 | 1 | 155169355 | 0.0172754 | 32072 | 0.156595 | 155169355 | 1.73E-69 | 1 | 343836 | 0.0020515 |
| 415 | rs4293567 | A | G | A | G | 0.020848 | -0.013002 | 0.31451 | 0.27805 | 2 | 18676203 | 0.0182453 | 32072 | 0.476083 | 18676203 | 1.42E-21 | 2 | 343836 | 0.002185 |
| 416 | rs429358 | C | T | C | T | -0.017723 | -0.039193 | 0.15624 | 0.1289 | 19 | 45411941 | 0.0252294 | 32072 | 0.12031 | 45411941 | 2.41E-10 | 19 | 343836 | 0.0027985 |
| 417 | rs4415952 | C | T | C | T | 0.014151 | -0.009297 | 0.73315 | 0.712849 | 14 | 73189153 | 0.0184264 | 32072 | 0.613889 | 73189153 | 7.56E-10 | 14 | 343836 | 0.0022994 |
| 418 | rs4441044 | A | G | A | G | 0.011656 | -0.007949 | 0.64433 | 0.688446 | 11 | 69500363 | 0.017774 | 32072 | 0.654693 | 69500363 | 3.87E-08 | 11 | 343836 | 0.0021204 |
| 419 | rs4468717 | T | C | T | C | -0.021382 | -0.088613 | 0.077674 | 0.0735041 | 18 | 3457606 | 0.0366182 | 32072 | 0.0155246 | 3457606 | 1.74E-08 | 18 | 343836 | 0.0037938 |
| 420 | rs45487598 | A | G | A | G | 0.018368 | -0.037241 | 0.11689 | 0.105964 | 10 | 16958653 | 0.0256658 | 32072 | 0.14678 | 16958653 | 7.71E-09 | 10 | 343836 | 0.0031807 |
| 421 | rs455213 | C | T | C | T | 0.018095 | 9.77E-05 | 0.4578 | 0.42941 | 5 | 34660235 | 0.0171955 | 32072 | 0.995469 | 34660235 | 7.82E-19 | 5 | 343836 | 0.0020416 |
| 422 | rs4575545 | A | G | A | G | -0.026075 | 0.0054874 | 0.30716 | 0.341184 | 16 | 79755446 | 0.0187737 | 32072 | 0.770064 | 79755446 | 4.47E-32 | 16 | 343836 | 0.0022117 |
| 423 | rs4693210 | G | A | G | A | -0.01573 | 0.0424735 | 0.43773 | 0.450038 | 4 | 89208565 | 0.0172812 | 32072 | 0.0139794 | 89208565 | 1.93E-14 | 4 | 343836 | 0.0020547 |
| 424 | rs4740156 | A | T | A | T | -0.011614 | -0.007698 | 0.57232 | 0.576189 | 9 | 134236538 | 0.0173397 | 32072 | 0.65708 | 134236538 | 1.70E-08 | 9 | 343836 | 0.0020591 |
| 425 | rs4744712 | C | A | C | A | 0.013584 | 0.0278443 | 0.60157 | 0.603128 | 9 | 71434707 | 0.0172968 | 32072 | 0.107443 | 71434707 | 6.14E-11 | 9 | 343836 | 0.0020769 |
| 426 | rs4751640 | C | A | C | A | -0.012706 | 0.001132 | 0.6938 | 0.67582 | 10 | 119572168 | 0.0183031 | 32072 | 0.950686 | 119572168 | 1.09E-08 | 10 | 343836 | 0.0022229 |
| 427 | rs478425 | T | G | T | G | -0.013937 | 0.0417907 | 0.36954 | 0.396413 | 1 | 234863602 | 0.0175495 | 32072 | 0.0172516 | 234863602 | 3.67E-11 | 1 | 343836 | 0.0021062 |
| 428 | rs4962687 | A | G | A | G | -0.016333 | -0.023757 | 0.68142 | 0.696662 | 10 | 126404141 | 0.018808 | 32072 | 0.206542 | 126404141 | 7.81E-14 | 10 | 343836 | 0.0021853 |
| 429 | rs4966019 | T | C | T | C | -0.033611 | -0.036471 | 0.6405 | 0.612286 | 15 | 99274326 | 0.0176201 | 32072 | 0.0384636 | 99274326 | 1.66E-56 | 15 | 343836 | 0.0021216 |
| 430 | rs508205 | A | G | A | G | 0.014351 | -0.018918 | 0.55818 | 0.581481 | 11 | 120057343 | 0.0172148 | 32072 | 0.271787 | 120057343 | 2.30E-12 | 11 | 343836 | 0.0020458 |
| 431 | rs527616 | G | C | G | C | -0.01258 | -0.025825 | 0.64222 | 0.639134 | 18 | 24337424 | 0.0175951 | 32072 | 0.142173 | 24337424 | 2.59E-08 | 18 | 343836 | 0.0022595 |
| 432 | rs538656 | T | G | T | G | 0.020552 | 0.0217002 | 0.23401 | 0.239005 | 18 | 57850422 | 0.0199489 | 32072 | 0.276688 | 57850422 | 1.08E-17 | 18 | 343836 | 0.0023993 |
| 433 | rs541091 | A | G | A | G | -0.013042 | 0.0048282 | 0.47483 | 0.475772 | 6 | 160770552 | 0.0168931 | 32072 | 0.775022 | 160770552 | 1.54E-10 | 6 | 343836 | 0.0020372 |
| 434 | rs541564 | A | G | A | G | 0.014458 | -0.012841 | 0.35411 | 0.417017 | 10 | 69848298 | 0.0181978 | 32072 | 0.480401 | 69848298 | 1.31E-11 | 10 | 343836 | 0.0021364 |
| 435 | rs55838345 | C | G | C | G | 0.017286 | -0.027978 | 0.12404 | 0.105106 | 10 | 82085120 | 0.0261749 | 32072 | 0.285125 | 82085120 | 2.40E-08 | 10 | 343836 | 0.0030975 |
| 436 | rs55902013 | G | C | G | C | -0.015633 | 0.0133942 | 0.21667 | 0.217385 | 6 | 39150657 | 0.0202123 | 32072 | 0.507539 | 39150657 | 2.35E-10 | 6 | 343836 | 0.0024671 |
| 437 | rs56379622 | A | G | A | G | -0.037405 | 0.0493917 | 0.0447 | 0.0483518 | 9 | 130756222 | 0.0366153 | 32072 | 0.177358 | 130756222 | 2.90E-14 | 9 | 343836 | 0.0049199 |
| 438 | rs57158761 | G | A | G | A | 0.013109 | 0.0026321 | 0.43663 | 0.44276 | 3 | 185371172 | 0.0173436 | 32072 | 0.879377 | 185371172 | 1.69E-10 | 3 | 343836 | 0.0020524 |
| 439 | rs580241 | A | G | A | G | -0.016036 | -0.017452 | 0.76343 | 0.747243 | 11 | 66066349 | 0.0197299 | 32072 | 0.376391 | 66066349 | 5.58E-11 | 11 | 343836 | 0.0024464 |
| 440 | rs603424 | A | G | A | G | 0.015215 | 0.0115228 | 0.168 | 0.204703 | 10 | 102075479 | 0.0206091 | 32072 | 0.576084 | 102075479 | 2.15E-08 | 10 | 343836 | 0.0027174 |
| 441 | rs6040060 | G | A | G | A | -0.014796 | 0.0240143 | 0.72089 | 0.752777 | 20 | 10640201 | 0.0203224 | 32072 | 0.237337 | 10640201 | 1.63E-10 | 20 | 343836 | 0.0023145 |
| 442 | rs60767324 | C | T | C | T | -0.020099 | 0.0231599 | 0.086706 | 0.073071 | 7 | 50732659 | 0.0329719 | 32072 | 0.482421 | 50732659 | 2.85E-08 | 7 | 343836 | 0.0036212 |
| 443 | rs6127099 | T | A | T | A | -0.015153 | -0.014565 | 0.27855 | 0.306196 | 20 | 52731402 | 0.0186351 | 32072 | 0.434465 | 52731402 | 6.21E-11 | 20 | 343836 | 0.0023174 |
| 444 | rs6142206 | A | G | A | G | 0.016467 | -0.049977 | 0.42115 | 0.387851 | 20 | 33212055 | 0.0171569 | 32072 | 0.0035805 | 33212055 | 1.28E-15 | 20 | 343836 | 0.002059 |
| 445 | rs62106258 | C | T | C | T | -0.034223 | -0.07713 | 0.048343 | 0.0414273 | 2 | 417167 | 0.0432784 | 32072 | 0.0747205 | 417167 | 5.12E-13 | 2 | 343836 | 0.0047384 |
| 446 | rs62294340 | A | G | A | G | -0.017802 | -0.014566 | 0.38339 | 0.35183 | 3 | 169155476 | 0.0178748 | 32072 | 0.415153 | 169155476 | 1.72E-17 | 3 | 343836 | 0.0020915 |
| 447 | rs62435145 | T | G | T | G | 0.033593 | -0.009943 | 0.69192 | 0.618224 | 7 | 1286567 | 0.018146 | 32072 | 0.583729 | 1286567 | 1.64E-49 | 7 | 343836 | 0.0022706 |
| 448 | rs62580785 | C | T | C | T | -0.014583 | 0.0197419 | 0.17947 | 0.165844 | 9 | 113051296 | 0.0214295 | 32072 | 0.356921 | 113050798 | 4.50E-08 | 9 | 343836 | 0.002666 |
| 449 | rs6499163 | T | G | T | G | -0.015671 | 0.0211087 | 0.17424 | 0.190594 | 16 | 68243486 | 0.0213775 | 32072 | 0.323433 | 68243486 | 4.81E-09 | 16 | 343836 | 0.002677 |
| 450 | rs6727096 | T | A | T | A | -0.013893 | -0.007997 | 0.20392 | 0.187908 | 2 | 161307677 | 0.0209464 | 32072 | 0.702637 | 161307677 | 4.25E-08 | 2 | 343836 | 0.0025351 |
| 451 | rs6760053 | G | C | G | C | -0.012718 | -0.035558 | 0.46536 | 0.41808 | 2 | 111932997 | 0.0173401 | 32072 | 0.0403032 | 111932997 | 4.41E-10 | 2 | 343836 | 0.0020385 |
| 452 | rs676015 | C | T | C | T | -0.014522 | 0.0018839 | 0.6312 | 0.647106 | 6 | 2064648 | 0.0181181 | 32072 | 0.917186 | 2064648 | 9.76E-12 | 6 | 343836 | 0.0021323 |
| 453 | rs6774307 | C | T | C | T | 0.023975 | -0.039863 | 0.077872 | 0.0881274 | 3 | 170759494 | 0.0286589 | 32072 | 0.164243 | 170759494 | 2.96E-10 | 3 | 343836 | 0.003805 |
| 454 | rs686364 | G | A | G | A | 0.018345 | -0.009625 | 0.2346 | 0.282008 | 21 | 31587793 | 0.0193135 | 32072 | 0.618237 | 31587793 | 1.88E-14 | 21 | 343836 | 0.0023952 |
| 455 | rs6965823 | A | C | A | C | -0.013374 | -0.014558 | 0.32213 | 0.330434 | 7 | 4703360 | 0.019256 | 32072 | 0.449628 | 4703360 | 7.21E-10 | 7 | 343836 | 0.0021705 |
| 456 | rs700750 | A | C | A | C | 0.014481 | -0.019098 | 0.62887 | 0.655402 | 7 | 46753491 | 0.0176078 | 32072 | 0.278081 | 46753491 | 5.67E-12 | 7 | 343836 | 0.0021024 |
| 457 | rs7093087 | A | G | A | G | 0.017763 | -0.057365 | 0.17448 | 0.179392 | 10 | 104700444 | 0.0218542 | 32072 | 0.008667 | 104700444 | 3.40E-11 | 10 | 343836 | 0.0026798 |
| 458 | rs7154553 | G | A | G | A | 0.017367 | -0.036309 | 0.19838 | 0.183828 | 14 | 102686183 | 0.020869 | 32072 | 0.0818842 | 102686183 | 1.04E-11 | 14 | 343836 | 0.0025535 |
| 459 | rs7161366 | C | G | C | G | -0.014603 | -0.0571 | 0.71131 | 0.725364 | 14 | 37930016 | 0.0186 | 32072 | 0.0021412 | 37930016 | 7.58E-11 | 14 | 343836 | 0.0022435 |
| 460 | rs7179427 | A | G | A | G | 0.016041 | -0.032364 | 0.72778 | 0.687913 | 15 | 72516821 | 0.0195376 | 32072 | 0.0976225 | 72516821 | 2.22E-12 | 15 | 343836 | 0.002285 |
| 461 | rs7224610 | A | C | A | C | -0.028799 | -0.026901 | 0.60224 | 0.60729 | 17 | 53364788 | 0.0176843 | 32072 | 0.128211 | 53364788 | 5.23E-43 | 17 | 343836 | 0.0020945 |
| 462 | rs72681698 | C | T | C | T | -0.071712 | -0.059385 | 0.011039 | 0.0067527 | 14 | 51207741 | 0.11229 | 32072 | 0.596905 | 51207741 | 1.64E-13 | 14 | 343836 | 0.0097228 |
| 463 | rs72799820 | T | C | T | C | -0.016962 | 0.0098002 | 0.14999 | 0.202504 | 16 | 71647450 | 0.0215821 | 32072 | 0.649765 | 71647450 | 2.70E-09 | 16 | 343836 | 0.0028513 |
| 464 | rs72818964 | A | G | A | G | 0.015535 | 0.0027786 | 0.17609 | 0.140415 | 2 | 101578022 | 0.0231824 | 32072 | 0.904595 | 101578022 | 5.59E-09 | 2 | 343836 | 0.0026653 |
| 465 | rs72951456 | T | C | T | C | 0.026435 | 0.0166218 | 0.048935 | 0.0418811 | 6 | 53832348 | 0.0416359 | 32072 | 0.689733 | 53832348 | 3.17E-08 | 6 | 343836 | 0.0047786 |
| 466 | rs7302925 | G | A | G | A | -0.021027 | -0.002495 | 0.80212 | 0.81198 | 12 | 56861458 | 0.0212431 | 32072 | 0.906522 | 56861458 | 1.48E-16 | 12 | 343836 | 0.0025463 |
| 467 | rs738408 | T | C | T | C | -0.019314 | -0.006322 | 0.21583 | 0.252562 | 22 | 44324730 | 0.020252 | 32072 | 0.754918 | 44324730 | 4.83E-15 | 22 | 343836 | 0.0024661 |
| 468 | rs74606487 | G | A | G | A | -0.016513 | -0.018128 | 0.15062 | 0.119077 | 16 | 89795305 | 0.0269305 | 32072 | 0.50085 | 89795305 | 1.31E-08 | 16 | 343836 | 0.0029051 |
| 469 | rs7461961 | A | G | A | G | 0.012278 | 0.0043146 | 0.54612 | 0.497453 | 8 | 77238737 | 0.0171135 | 32072 | 0.800953 | 77222269 | 2.57E-09 | 8 | 343836 | 0.0020611 |
| 470 | rs75588192 | A | G | A | G | 0.023169 | -0.018236 | 0.13826 | 0.140065 | 12 | 133048600 | 0.0253548 | 32072 | 0.471992 | 133048600 | 9.96E-15 | 12 | 343836 | 0.0029934 |
| 471 | rs75964023 | T | C | T | C | -0.14547 | -0.04252 | 0.039752 | 0.107415 | 4 | 9979159 | 0.0410042 | 32072 | 0.299754 | 9979159 | 1.41E-170 | 4 | 343836 | 0.005222 |
| 472 | rs7616014 | C | G | C | G | 0.013648 | 0.0156985 | 0.78126 | 0.724909 | 3 | 114427057 | 0.0202888 | 32072 | 0.439076 | 114427057 | 3.59E-08 | 3 | 343836 | 0.0024769 |
| 473 | rs76358556 | G | A | G | A | -0.021554 | -0.000438 | 0.20379 | 0.184184 | 3 | 126014827 | 0.0220599 | 32072 | 0.984165 | 126014827 | 1.28E-17 | 3 | 343836 | 0.0025222 |
| 474 | rs76895963 | G | T | G | T | -0.055657 | 0.195316 | 0.021069 | 0.0159991 | 12 | 4384844 | 0.0667339 | 32072 | 0.0034248 | 4384844 | 9.76E-13 | 12 | 343836 | 0.0078016 |
| 475 | rs7696556 | C | A | C | A | -0.017419 | 0.0399084 | 0.1395 | 0.227587 | 4 | 146719953 | 0.0238817 | 32072 | 0.0947044 | 146719953 | 3.02E-09 | 4 | 343836 | 0.0029372 |
| 476 | rs7736102 | A | G | A | G | 0.014671 | 0.0367709 | 0.56872 | 0.477901 | 5 | 131604257 | 0.0170096 | 32072 | 0.0306351 | 131604257 | 9.15E-13 | 5 | 343836 | 0.0020538 |
| 477 | rs77542162 | G | A | G | A | -0.053442 | 0.110878 | 0.022979 | 0.0135945 | 17 | 67081278 | 0.0793593 | 32072 | 0.162363 | 67081278 | 3.49E-15 | 17 | 343836 | 0.0067886 |
| 478 | rs7779637 | G | A | G | A | -0.014326 | 0.0205421 | 0.5475 | 0.599434 | 7 | 128742164 | 0.0170489 | 32072 | 0.228246 | 128742164 | 2.64E-12 | 7 | 343836 | 0.0020478 |
| 479 | rs78177245 | T | C | T | C | -0.035338 | 0.0842139 | 0.027568 | 0.0214147 | 4 | 11311285 | 0.0595245 | 32072 | 0.157134 | 11311285 | 1.16E-08 | 4 | 343836 | 0.0061939 |
| 480 | rs784257 | C | T | C | T | 0.014875 | -0.00602 | 0.81295 | 0.835634 | 18 | 53397199 | 0.021491 | 32072 | 0.779394 | 53397199 | 1.34E-08 | 18 | 343836 | 0.0026187 |
| 481 | rs78671965 | T | A | T | A | 0.027075 | -0.014995 | 0.055477 | 0.0530656 | 17 | 7859317 | 0.0455121 | 32072 | 0.741793 | 7859317 | 1.73E-09 | 17 | 343836 | 0.0044965 |
| 482 | rs79239275 | A | C | A | C | 0.027231 | -0.015251 | 0.045961 | 0.0397583 | 3 | 132491193 | 0.0438625 | 32072 | 0.728071 | 132491193 | 2.06E-08 | 3 | 343836 | 0.0048563 |
| 483 | rs7952403 | G | A | G | A | -0.017187 | 0.0106259 | 0.87396 | 0.884781 | 11 | 111324632 | 0.0270942 | 32072 | 0.694922 | 111324632 | 2.02E-08 | 11 | 343836 | 0.0030633 |
| 484 | rs8039645 | A | C | A | C | 0.014158 | 0.0177824 | 0.2265 | 0.243478 | 15 | 90670222 | 0.0200626 | 32072 | 0.375433 | 90670222 | 1.05E-08 | 15 | 343836 | 0.0024744 |
| 485 | rs807624 | T | G | T | G | -0.018735 | 0.0223474 | 0.35776 | 0.413239 | 2 | 15782471 | 0.0178544 | 32072 | 0.210701 | 15782471 | 9.06E-19 | 2 | 343836 | 0.0021177 |
| 486 | rs833805 | G | A | G | A | 0.026432 | 0.0289641 | 0.8848 | 0.887139 | 6 | 44030011 | 0.0269655 | 32072 | 0.28277 | 44030011 | 2.40E-15 | 6 | 343836 | 0.0033377 |
| 487 | rs836968 | T | C | T | C | -0.014747 | 0.0433227 | 0.27078 | 0.298592 | 12 | 50267335 | 0.0195741 | 32072 | 0.0268794 | 50267335 | 1.56E-10 | 12 | 343836 | 0.0023044 |
| 488 | rs854917 | T | C | T | C | -0.012795 | -0.031308 | 0.73758 | 0.748695 | 6 | 90127390 | 0.019705 | 32072 | 0.112099 | 90127390 | 3.60E-08 | 6 | 343836 | 0.0023222 |
| 489 | rs871375 | A | G | A | G | -0.018139 | -0.023745 | 0.65649 | 0.705976 | 2 | 242421866 | 0.0180506 | 32072 | 0.188352 | 242421866 | 2.33E-17 | 2 | 343836 | 0.0021399 |
| 490 | rs9297949 | C | A | C | A | 0.020148 | -0.048334 | 0.52978 | 0.542039 | 8 | 95969445 | 0.017033 | 32072 | 0.0045442 | 95969445 | 4.05E-23 | 8 | 343836 | 0.0020344 |
| 491 | rs9534949 | G | C | G | C | -0.015739 | 0.0411691 | 0.72989 | 0.720995 | 13 | 48654455 | 0.0192898 | 32072 | 0.0328231 | 48654455 | 5.54E-12 | 13 | 343836 | 0.002284 |
| 492 | rs9599875 | T | A | T | A | -0.017061 | 0.0187571 | 0.30319 | 0.257883 | 13 | 72342035 | 0.0191043 | 32072 | 0.326184 | 72342035 | 1.30E-14 | 13 | 343836 | 0.0022139 |
| 493 | rs9761429 | G | A | G | A | -0.034065 | -0.044134 | 0.24828 | 0.267476 | 4 | 9553433 | 0.0207051 | 32072 | 0.0330438 | 9530799 | 1.28E-45 | 4 | 343836 | 0.0024025 |
| 494 | rs9807214 | A | G | A | G | 0.012822 | 0.0400078 | 0.30265 | 0.261208 | 18 | 42782952 | 0.018628 | 32072 | 0.0317366 | 42782952 | 7.07E-09 | 18 | 343836 | 0.0022147 |
| 495 | rs9932625 | A | G | A | G | 0.019386 | 0.0023347 | 0.22886 | 0.253973 | 16 | 51735746 | 0.0199883 | 32072 | 0.907014 | 51735746 | 1.25E-15 | 16 | 343836 | 0.0024234 |
