# Supplementary material for: Exploring the link between serum uric acid and colorectal cancer: Insights from genetic evidence and observational data
Source: Medicine (Baltimore). 2024 Nov 22;103(47):e40591. doi: 10.1097/MD.0000000000040591 (PMC11596604; doi:10.1097/MD.0000000000040591)
Supplement: SUPPLEMENTARY MATERIAL [file medi-103-e40591-s002.docx]

| **Supplementary Table S2. Characteristics of the NHANES cohort.** | | | | |
| --- | --- | --- | --- | --- |
| **Characteristics** | **Live (N=151)** | **Dead (N=141)** | **Total(N=292)** | **p-value** |
| **Smoking** |  |  |  | 0.51 |
| Former | 65(22.26%) | 69(23.63%) | 134(45.89%) |  |
| Never | 69(23.63%) | 55(18.84%) | 124(42.47%) |  |
| Current | 17(5.82%) | 17(5.82%) | 34(11.64%) |  |
| **Serum urate** |  |  |  |  |
| Mean±SD | 5.86±1.42 | 6.00±1.55 | 5.93±1.48 |  |
| Median[min-max] | 5.60[2.60,10.90] | 5.90[2.40,12.20] | 5.80[2.40,12.20] |  |
| **Age** |  |  |  |  |
| Mean±SD | 67.52±11.39 | 75.15±9.24 | 71.21±11.07 |  |
| Median[min-max] | 70.00[22.00,84.00] | 79.00[37.00,85.00] | 74.00[22.00,85.00] |  |
| **Gender** |  |  |  | 0.15 |
| Female | 79(27.05%) | 61(20.89%) | 140(47.95%) |  |
| Male | 72(24.66%) | 80(27.40%) | 152(52.05%) |  |
| **Race** |  |  |  | 0.03 |
| Mexican American | 16(5.48%) | 5(1.71%) | 21(7.19%) |  |
| Non-Hispanic Black | 29(9.93%) | 23(7.88%) | 52(17.81%) |  |
| Non-Hispanic White | 88(30.14%) | 104(35.62%) | 192(65.75%) |  |
| Other Hispanic | 10(3.42%) | 6(2.05%) | 16(5.48%) |  |
| Other Race | 8(2.74%) | 3(1.03%) | 11(3.77%) |  |
| **Marry** |  |  |  | 0.26 |
| Divorced | 21(7.24%) | 14(4.83%) | 35(12.07%) |  |
| Living with partner | 1(0.34%) | 1(0.34%) | 2(0.69%) |  |
| Married | 78(26.90%) | 71(24.48%) | 149(51.38%) |  |
| Never married | 9(3.10%) | 7(2.41%) | 16(5.52%) |  |
| Separated | 9(3.10%) | 3(1.03%) | 12(4.14%) |  |
| Widowed | 32(11.03%) | 44(15.17%) | 76(26.21%) |  |
| **Poverty** |  |  |  |  |
| Mean±SD | 2.54±1.58 | 2.45±1.49 | 2.49±1.53 |  |
| Median[min-max] | 2.19[0.0e+0,5.00] | 2.11[0.05,5.00] | 2.17[0.0e+0,5.00] |  |
| **Education** |  |  |  | 5.80E-07 |
| 9-11th Grade (Includes 12th grade with no diploma) | 13(4.47%) | 21(7.22%) | 34(11.68%) |  |
| 9-11th grade (Includes 12th grade with no diploma) | 8(2.75%) | 6(2.06%) | 14(4.81%) |  |
| College Graduate or above | 12(4.12%) | 13(4.47%) | 25(8.59%) |  |
| College graduate or above | 10(3.44%) | 6(2.06%) | 16(5.50%) |  |
| High School Grad/GED or Equivalent | 19(6.53%) | 23(7.90%) | 42(14.43%) |  |
| High school graduate/GED or equivalent | 24(8.25%) | 2(0.69%) | 26(8.93%) |  |
| Less Than 9th Grade | 9(3.09%) | 26(8.93%) | 35(12.03%) |  |
| Less than 9th grade | 12(4.12%) | 4(1.37%) | 16(5.50%) |  |
| Some College or AA degree | 15(5.15%) | 29(9.97%) | 44(15.12%) |  |
| Some college or AA degree | 28(9.62%) | 11(3.78%) | 39(13.40%) |  |
