# Supplementary figures and images for: Exploring the link between serum uric acid and colorectal cancer: Insights from genetic evidence and observational data
Source: Medicine (Baltimore). 2024 Nov 22;103(47):e40591. doi: 10.1097/MD.0000000000040591 (PMC11596604; doi:10.1097/MD.0000000000040591)

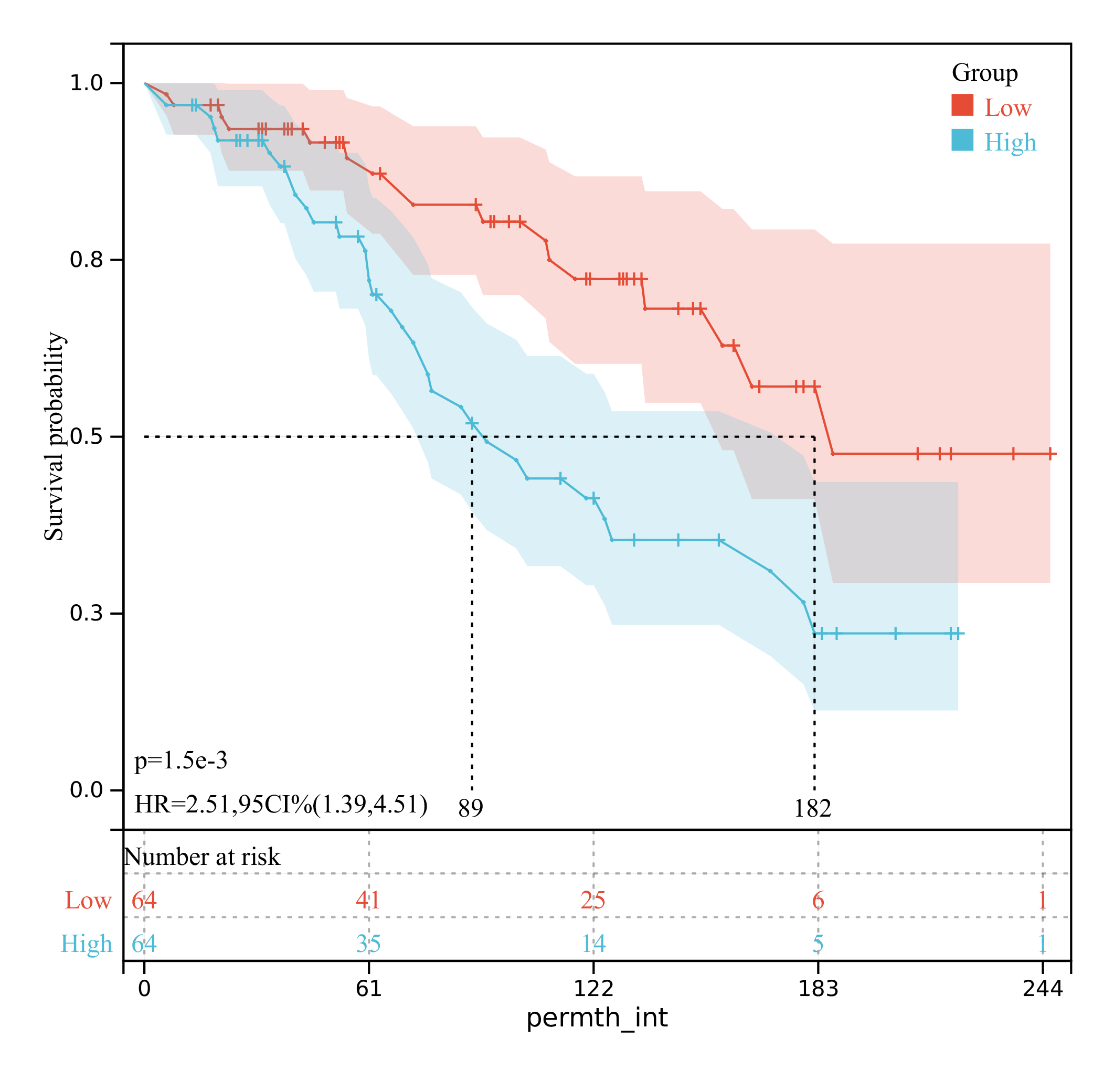

Supplement: SUPPLEMENTARY MATERIAL [file medi-103-e40591-s003.tif]
